# Myricetin protects *Galleria mellonella* against *Staphylococcus aureus* infection and inhibits multiple virulence factors

**DOI:** 10.1038/s41598-017-02712-1

**Published:** 2017-06-06

**Authors:** L. N. Silva, G. C. A. Da Hora, T. A. Soares, M. S. Bojer, H. Ingmer, A. J. Macedo, D. S. Trentin

**Affiliations:** 1 Centro de Biotecnologia do Estado do Rio Grande do Sul, Porto Alegre, Universidade Federal do Rio Grande do Sul, Porto Alegre-RS, 91501-970 Brazil; 2 Faculdade de Farmácia, Universidade Federal do Rio Grande do Sul, Porto Alegre-RS, 90610-000 Brazil; 3 Departmento de Química Fundamental, Universidade Federal de Pernambuco, Recife-PE, 50670-901 Brazil; 4 Department of Veterinary and Animal Sciences, Faculty of Health and Medical Sciences, University of Copenhagen, Frederiksberg C, 1870 Denmark; 5 Department of Chemistry, Umeå University, 90187 Umeå, Sweden; 6 Departamento de Ciências Básicas da Saúde, Universidade Federal de Ciências da Saúde de Porto Alegre, Porto Alegre-RS, 90050-170 Brazil

## Abstract

*Staphylococcus aureus* is an opportunistic pathogen related to a variety of life-threatening infections but for which antimicrobial resistance is liming the treatment options. We report here that myricetin, but not its glycosylated form, can remarkably decrease the production of several *S. aureus* virulence factors, including adhesion, biofilm formation, hemolysis and staphyloxanthin production, without interfering with growth. Myricetin affects both surface proteins and secreted proteins which indicate that its action is unrelated to inhibition of the *agr* quorum sensing system. Analysis of virulence related gene expression and computational simulations of pivotal proteins involved in pathogenesis demonstrate that myricetin downregulates the *saeR* global regulator and interacts with sortase A and α-hemolysin. Furthermore, Myr confers a significant degree of protection against staphylococcal infection in the *Galleria mellonella* model. The present findings reveal the potential of Myr as an alternative multi-target antivirulence candidate to control *S. aureus* pathogenicity.

## Introduction

*Staphylococcus aureus* is an important human opportunistic pathogen involved in a wide range of human infections. This bacterium is frequently associated with bacteremia and infective endocarditis as well as osteoarticular, skin and soft tissue, pleuropulmonary and device-related infections^1^. Usage of bactericidal compounds has led to the emergence of several multidrug resistant strains; thus, making treatment of *S. aureus* infections a major challenge, especially in hospital settings^2, 3^. Moreover, the shortage of new antimicrobials coming to the market contributes to the low number of effective agents for some life-threatening infections^4^.

The pathogenicity of *S. aureus* is associated with the secretion of an impressive collection of virulence factors such as exotoxins and enzymes- hemolysin, enterotoxins and coagulase^5, 6^, biofilm formation^7^, staphyloxanthin pigment production^8^ and bacterial quorum sensing^9^. The α-hemolysin (Hla), most aptly referred to as α-toxin is secreted by most pathogenic *S. aureus* strains as a 33.2 kDa water-soluble monomer^10, 11^. Through binding to the host membrane, the monomer oligomerizes to form a 232.4 kDa membrane-inserted heptamer. This pore-forming toxin has been reported as an important protein that mediates tissue damage promoted by *S. aureus*. Other virulence factors are covalently anchored to the peptidoglycan by sortase enzymes, a group of cysteine transpeptidases widely distributed in Gram-positive bacteria^12^. Sortases, particularly SrtA, are essential for the functional assembly of surface proteins involved in staphylococcal adherence to host tissue and as such central in both systemic and localized infections^13, 14^.

**Figure 1:**
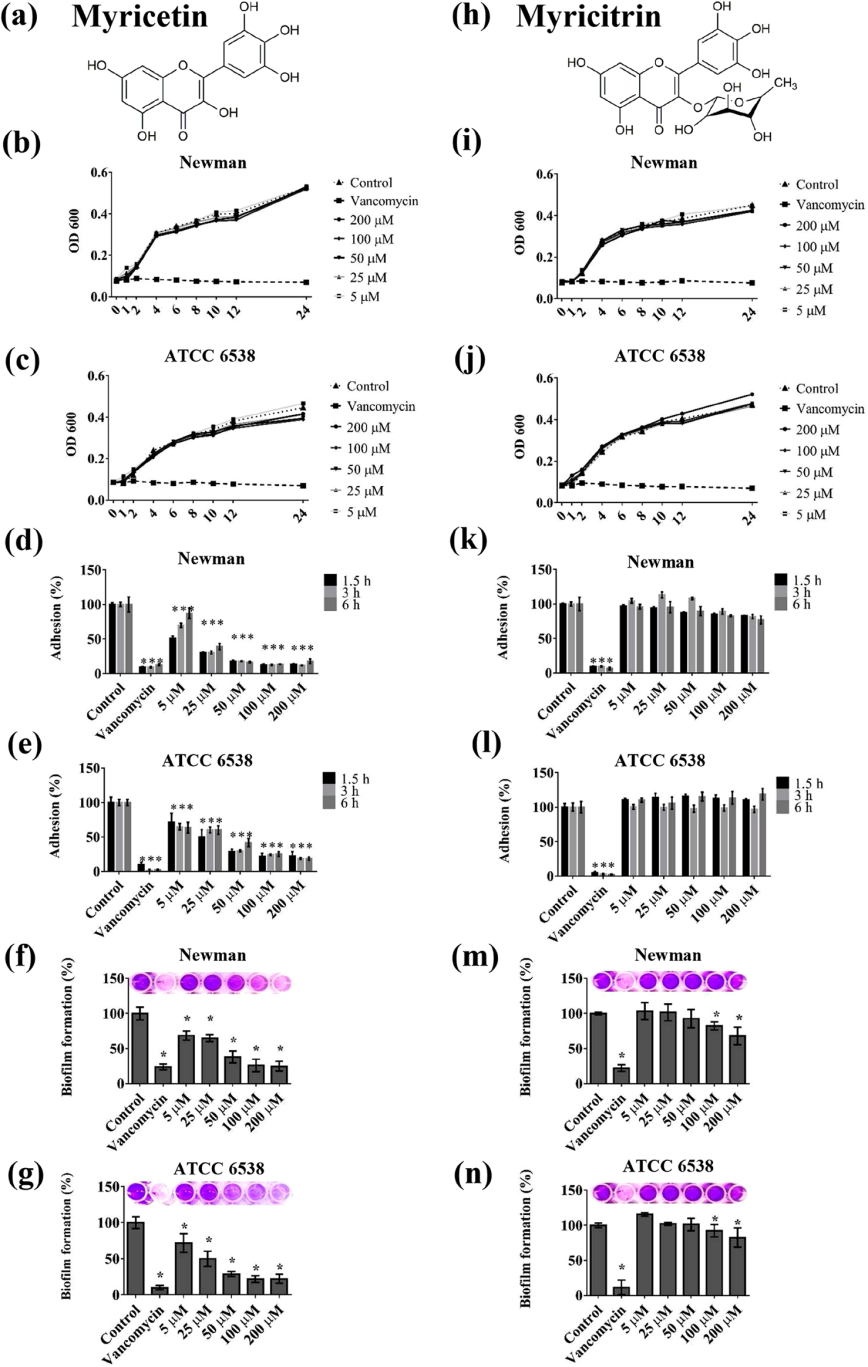
Effects of Myricetin (Myr) and Myricitrin (Myr-gly) on *S. aureus* growth, initial adhesion and biofilm formation. (**a**) Chemical structure of the Myr consisting of 3-hydroxyflavone backbone and 6 hydroxyl groups. (**b**,**c**) Growth kinetics at different concentrations of Myr (0, 5, 25, 50, 100 and 200 µM) during 24 h against *S. aureus* Newman and ATCC 6538. (**d**,**e**) Initial adherence of *S. aureus* Newman and ATCC 6538 treated with Myr during 1.5, 3 and 6 h. (**f**,**g**) Dose-response curve of biofilm formation tested against *S. aureus* Newman and ATCC 6538 in the presence of Myr. (**h**) Chemical structure of the flavonol Myr-gly, corresponding to derivative 3-O-rhamnoside of myricetin. (**i**,**j**) Growth kinetics at different concentrations of Myr-gly (0, 5, 25, 50, 100 and 200 µM) during 24 h against *S. aureus* Newman and ATCC 6538. (**k**,**l**) Initial adherence of *S. aureus* Newman and ATCC 6538 treated with Myr-gly during 1:30, 3 and 6 h. (**m**,**n**) Dose-response curve of biofilm formation tested against *S. aureus* Newman and ATCC 6538 in the presence of Myr-gly. *Represents statistically significant differences (*p*-value < 0.01) in relation to the control samples. Photos of crystal violet assay: increasing violet color indicates higher biofilm formation.

Hence, alternative therapeutic strategies involving antivirulence compounds have attracted great attention. Unlike antibacterials that aim to inhibit cell growth, antivirulence therapies are based on the inhibition of bacterial virulence. Importantly, virulence factors display a pathological role in bacterial colonization and invasion and are not essential for survival^15, 16^. In this regard, antivirulence therapies present a number of advantages since it could (i) produce a mild evolutionary pressure for development of resistance, (ii) provide an increased repertoire of pharmacological targets and (iii) generate agents with new mechanisms of action. Plants represent a rich source of bioactive molecules and thus, they are being explored for discovery and development of novel antivirulence agents. Among them, phenolic compounds such as flavonoids deserve special attention regarding their potential to control bacterial virulence^17^. Myricetin (Myr), 3,5,7,3′,4′,5′-hexahydroxyflavone (Fig. 1a), is a flavonoid commonly ingested through human diets such as fruits, vegetables, tea, berries and red wine. This flavonol has been proven to possess various beneficial pharmacological properties, including anti-oxidative and cytoprotective effects, anti-carcinogenic actions, antiviral properties as well as antiplatelet, anti-inflammation and anti-hyperlipidemia activities^18, 19^. In this study, we examined the effect of Myr and of its glycosylated form, myricitrin (Myr-gly- Fig. 1h), on several virulence factors produced by *S. aureus* and the potencial of the flavonol to protect the host during infection using the *in vivo Galleria mellonella* model.

## Results

### Initial adhesion and biofilm development in presence of Myr and Myr-gly

**Figure 2:**
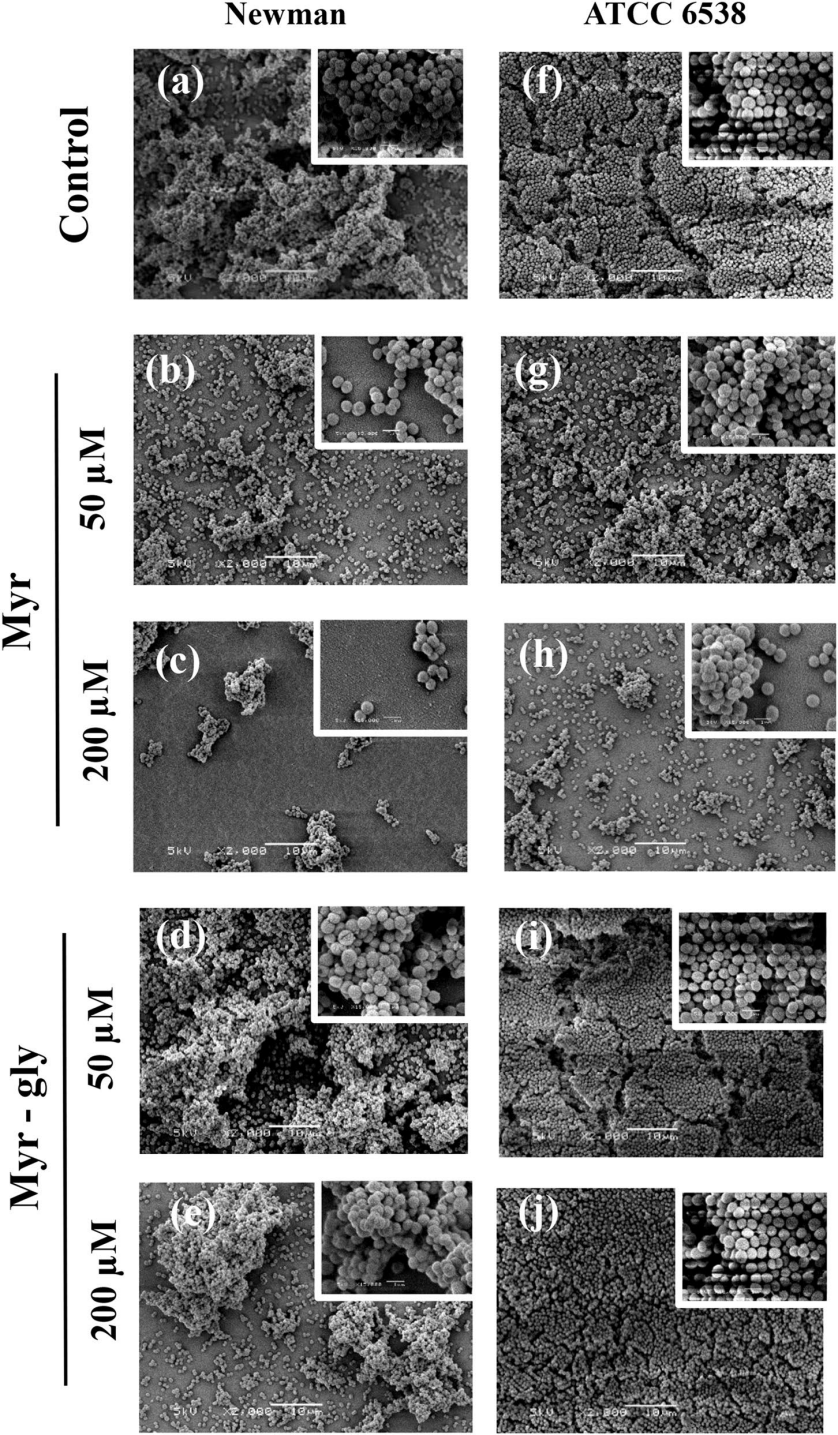
Scanning electron microscopy (SEM) images of biofilms. (**a–c**) *S. aureus* Newman treated with 0, 50 and 200 µM of Myr. **(d**,**e**) *S. aureus* Newman treated with 50 and 200 µM of Myr-gly. **(f–h**) *S. aureus* ATCC 6538 treated with 0, 50 and 200 µM of Myr. (**i**,**j**) *S. aureus* ATCC 6538 treated with 50 and 200 µM of Myr-gly. Scale bars: 10 µm (inserts in the images represent 1 µm).

To investigate whether Myr and Myr-gly hinder *S. aureus* biofilm formation, we evaluated distinct concentrations of the two compounds. Myr significantly inhibited *S. aureus* adhesion when tested in the early times of incubation (Fig. 1d,e) and biofilm development in a dose-dependent manner (Fig. 1f,g) without affecting bacterial growth (Fig. 1b,c). SEM images showed decreased biofilm formation on a hydrophobic polystyrene surface when *S. aureus* cells were exposed to Myr in comparison to untreated cells. These images correlated with the dose-response curve showing that Myr inhibited biofilm formation and kept most cells in the planktonic state (Fig. 2a–c and f–h). However, the same inhibition profile was not observed when biofilms were exposed to Myr-gly. In fact, Myr-gly did not affect *S. aureus* bacterial growth (Fig. 1i,j) and also was not able to avoid initial bacterial adhesion (Fig. 1k,l). Furthermore, Myr-gly showed a minor antibiofilm activity only at higher concentrations (Fig. 1m,n). SEM images indicated that cells in presence of Myr-gly (Fig. 2d,e and i,j) were similar to the control (Fig. 2a and f). In addition, microscopy evaluations also confirm that both compounds did not affect cell morphology, as shown in image inserts, confirming that their action is not related to cell death (Fig. 2). When tested against Gram-negative strains of *Pseudomonas aeruginosa* and *Klebsiella pneumoniae*, both compounds could not prevent biofilm formation (Supplementary Fig. S1).

### Adherence to cell-matrix protein and microbial surface hydrophobicity index in presence of Myr and Myr-gly

**Figure 3:**
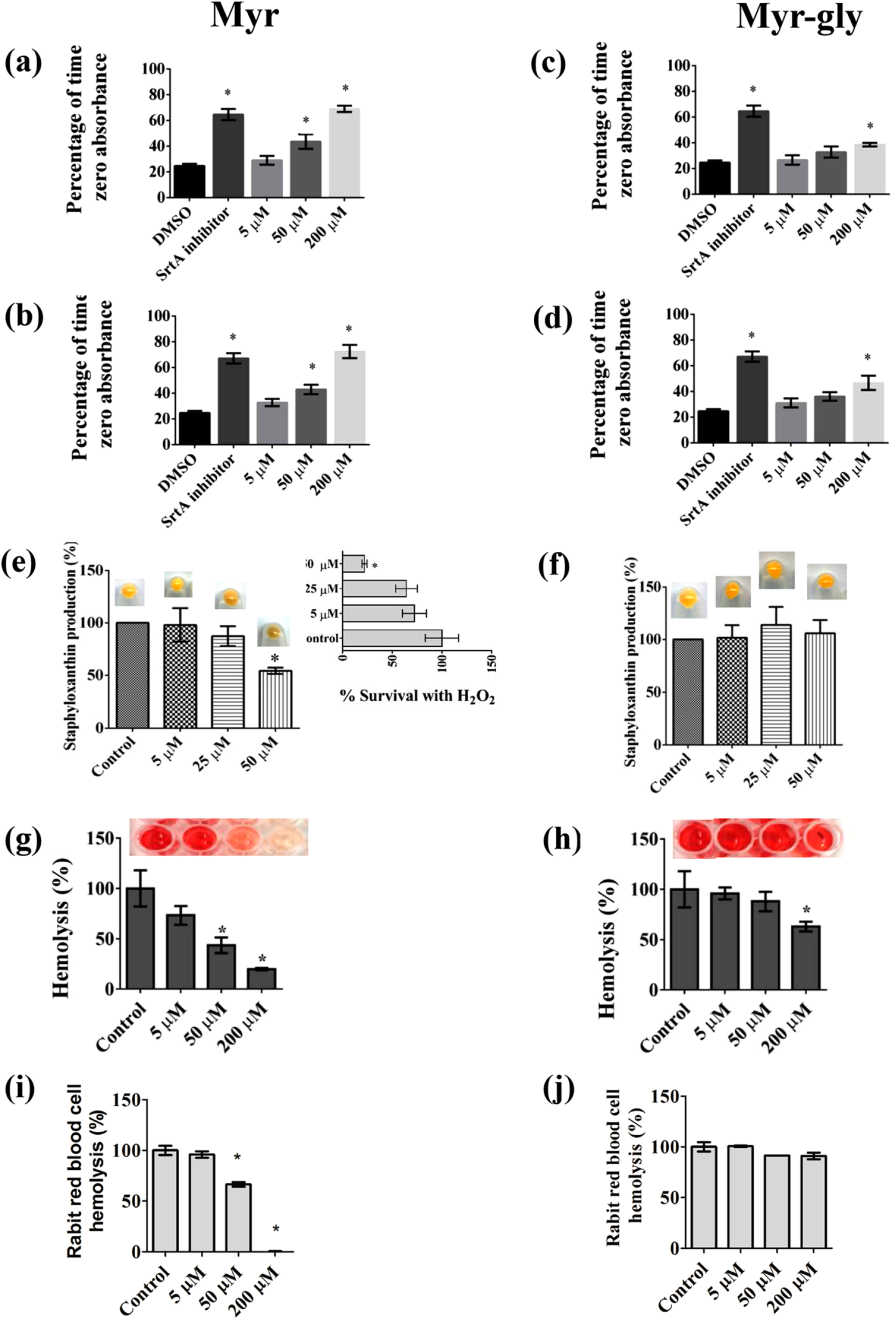
Effects of Myr and Myr-gly on *S. aureus* virulence factors: clumping, cell surface hydrophobicity, staphyloxanthin and hemolysis. (**a,b**) Inhibitory activity of Myr on the ability of *S. aureus* Newman and ATCC 6538 to clump with fibrinogen. (**c**,**d**) Inhibitory activity of Myr-gly on the ability of *S. aureus* Newman and ATCC 6538 to clump with fibrinogen. (**e**) Activity of Myr on the staphyloxanthin pigment production by *S. aureus* ATCC 6538. The insert present *S. aureus* ATCC 6538 susceptibility to H_2_O_2_ after staphyloxanthin reduction induced by Myr. (**f**) Activity of Myr-gly on the staphyloxanthin pigment production by *S. aureus* ATCC 6538. (**g**) Hemolysis promoted by supernatants of Myr treated-*S. aureus* ATCC 29213. (**h**) Hemolysis promoted by supernatants of Myr-gly treated-*S. aureus* ATCC 29213. (**i**) Effect of Myr on purified Hla induced hemolysis. (**j**) Effect of Myr-gly on purified Hla induced hemolysis. *Represents statistically significant differences (*p*-value < 0.01) in comparison to the control. The experiments were done in triplicate and representative images are shown.

An active sortase enzyme is indispensable for the adherence of *S. aureus* to host cell matrices and establishment of an infection. Proteins such as protein A, clumping factor proteins, and fibronectin-binding proteins are attached to the cell wall by this enzyme^12^. The clumping-inhibitory activity of Myr and Myr-gly was investigated against *S. aureus* strains. The treatment with Myr reduced the capacity of the bacteria to form clumps with fibrinogen in a dose-dependent manner. At 200 µM of Myr, *S. aureus* cells exhibited a threefold decrease in the level of fibrinogen cell clumping (Fig. 3a,b), similarly as the known-sortase A inhibitor quercetin. Conversely, Myr-gly treatment led to minor clumping-inhibitory effects only at higher dose (Fig. 3c,d).

The hydrophobic character of the bacterial surface has also been reported to play an important role in microbial attachment, not only to host cell matrices but also to abiotic surfaces^20^. Thus, cell surface hydrophobicity is relevant to the adhesion property of biofilm-producing bacteria, since it is known that hydrophobic cells adhere to a greater extent than hydrophilic cells. Myr was able to reduce the hydrophobicity of the surface of *S. aureus*, decreasing its hydrophobic nature by at least one-fold when compared to untreated controls (Supplementary Fig. S2).

### Staphyloxanthin production and hydrogen peroxide resistance in presence of Myr and Myr-gly

The golden pigment staphyloxanthin can be visually identified in the cell pellets of *S. aureus*. Cell pellets recovered from Myr-treated *S. aureus* clearly indicated that staphyloxanthin production was reduced compared to untreated cells. Quantitative analysis also showed that Myr (Fig. 3e), in contrast to Myr-gly (Fig. 3f), significantly decreased the staphyloxanthin production by *S. aureus*. Staphyloxanthin acts as an antioxidant by enabling the detoxification of host-immune system-generated reactive oxygen species such as oxygen radical (O_2_^−^) and hydrogen peroxide (H_2_O_2_). Therefore, we examined the effect of Myr on the survival rate of *S. aureus* in the presence of H_2_O_2_. Notably, Myr-treated cells were more susceptible to H_2_O_2_ than non-treated *S. aureus* (Fig. 3e insert).

### Cytotoxicity evaluation of Myr and Myr-gly to human erythrocytes and their capacity to prevent hemolysis caused by *S. aureus*

The possible cytotoxicity of Myr and Myr-gly was initially assessed using human erythrocytes. In this assay, all tested concentration of Myr and Myr-gly did not cause any damage to the erythrocyte membrane, unlike the positive control (Triton X-100) (Supplementary Fig. S3a). Following this, we investigated the effect of Myr and Myr-gly on hemolysis induced by *S. aureus* ATCC 29213 culture supernatants. The supernatant of Myr-treated bacteria clearly showed reduced hemolysis rates of human red blood cells in a dose-dependent manner (Fig. 3g), which was not oberved by Myr-gly treatment (Fig. 3h). Additionally, when Myr was mixed with purified Hla, the hemolytic activity was completely attenuated at 200 uM, a phenotype not observed for Myr-gly (Fig. 3i,j). Therefore, it is reasonable to presume that Myr directly interacts with the Hla protein.

### Transcriptional profiles of *S. aureus* cells in the presence of Myr or Myr-gly

**Figure 4:**
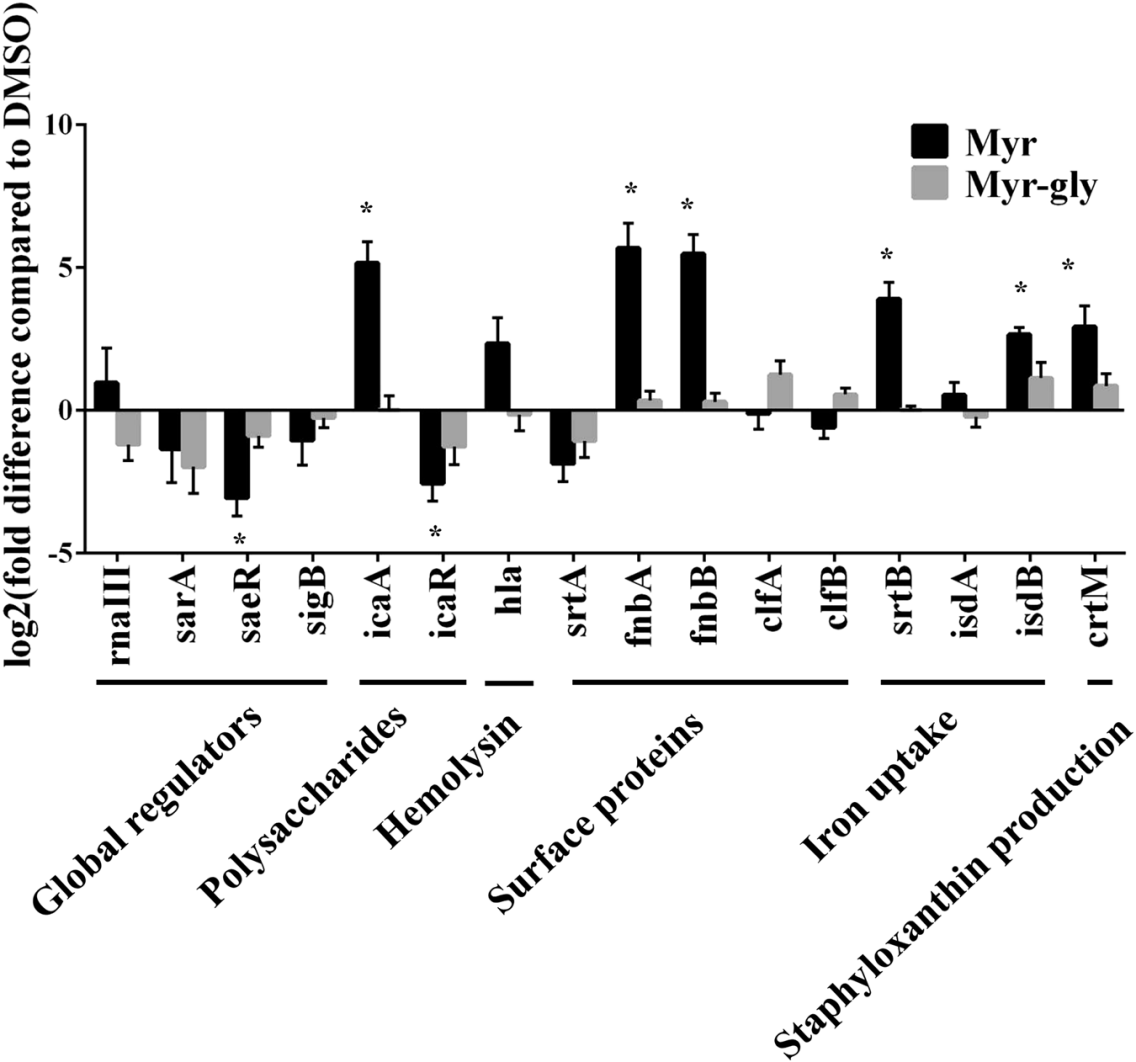
Transcriptional profile of *S. aureus* virulence genes upon treatment with Myr or Myr-gly. Transcript abundance was determined by qRT-PCR on cells grown for 2.5 h in BHI. The data are based on biological triplicates and the expression levels depicted are the mean log2 (fold difference) and standard deviations. *Represents significance level of *p* ≤ 0.02. Methodology can be found in Supplementary Methods.

To investigate the mechanism by which Myr attenuates a series of *S. aureus* virulence factors, real-time qRT-PCR was used to determine differential expression of virulence factor-related genes, including: global regulators (*rna*III, *sar*A, *sig*B and *sae*R), surface proteins (*fnb*A, *fnb*B, *clf*A and *clf*B), sortase proteins (*srt*A and *srt*B), iron uptake (*isd*A and *isd*B), polysaccharide production (*ica*A and *ica*R), hemolysin (*hla*) and staphyloxanthin production (*crt*M) (Fig. 4). While Myr-gly treatment resulted only in log2(fold difference) values below 2, Myr treatment caused significant up-regulation of *srt*B (approx. 15-fold, *p* = 0.0073) and *ica*A, *fnb*A and *fnb*B (40–50 fold, all *p* < 0.01). The increased expression of *ica*A was mirrored by a repression of *ica*R (approx. 6-fold, *p* = 0.0199). Moreover, Myr increased the expression of *isd*B (approx. 6-fold, *p* = 0.0027) and *crt*M (approx. 7-fold, *p* = 0.0203) and decreased *saeR* expression (approx. 8-fold, *p* = 0.0138). We observed only minor and not significant effects of the compounds on the *hla* (*p* = 0.0469) and *rna*III (0.3022) transcripts.

### Structural models for the Myr and SrtA complex

**Figure 5:**
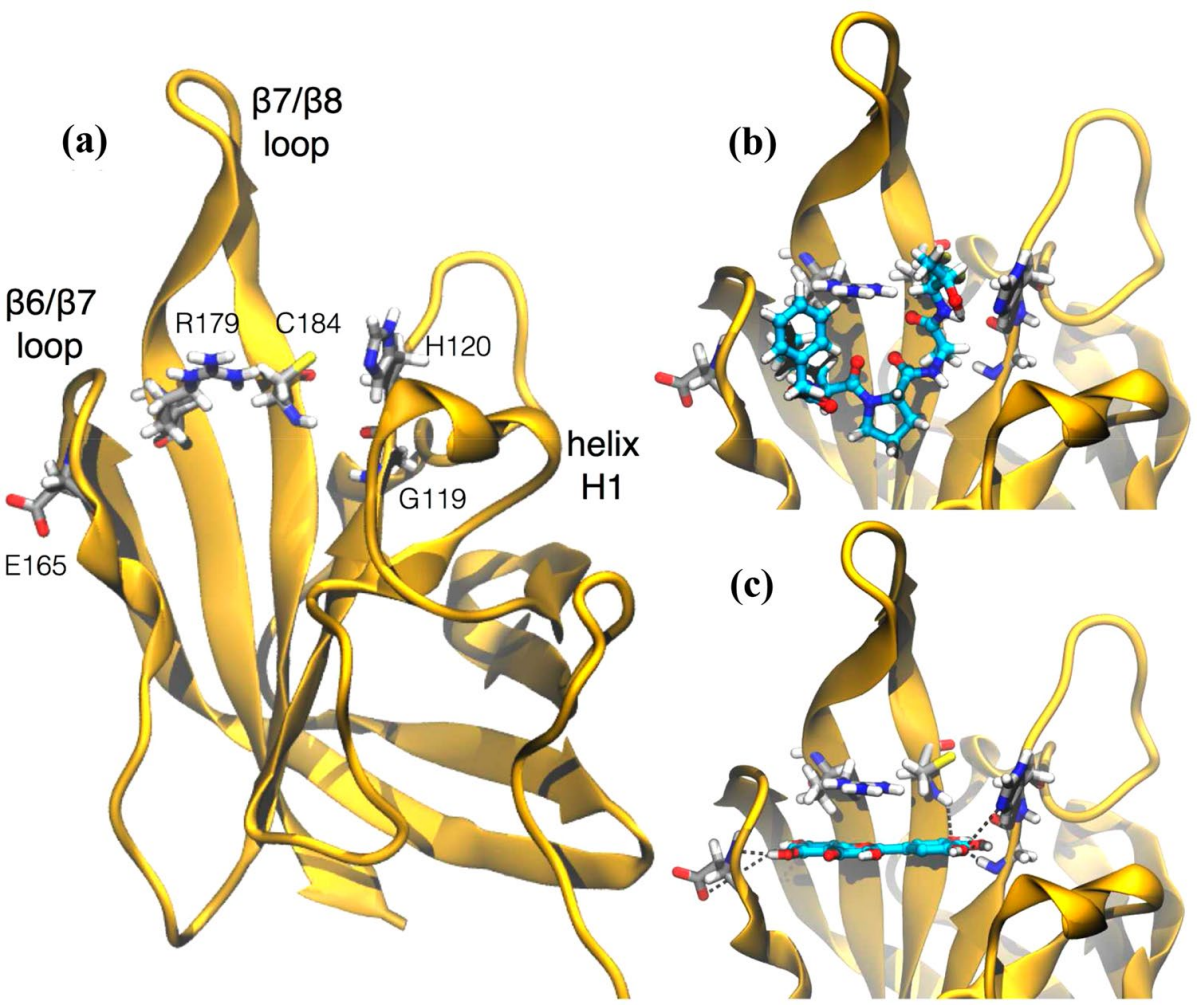
Computational studies of the structural model for the complex Myr-sortase A (SrtA). (**a**) Overall fold of SrtA highlighting regions undergoing large substrate-induced conformational changes. (**b**) Detail of the LPXTG sequence analog covalently bound to SrtA. (**c**) Lowest energy conformation of Myr representative of the lowest energy and most populated conformational cluster obtained through molecular docking calculations. Receptor residues are represented in gray and ligands in cyan. Potential hydrogen-bond interactions are shown in dashed black lines. Methodology can be found in Supplementary Methods.

Based on the findings that Myr suppress the clumping ability of *S. aureus* and that *srtA* expression was not significantly modulated, we investigated if Myr may interfere with sortase activity by directly binding to the SrtA protein. The NMR-derived structure of SrtA is covalently bound to an analog of the LPXTG sequence, its natural substrate. The substrate LPXTG binds to SrtA through a large groove that leads into the active site. The groove floor is formed by residues in strands 4 and 7 (groove floor), whereas the groove walls are formed by surface loops connecting strand 6 to strand 7 (β6/β7 loop), strand 7 to strand 8 (β7/β8 loop), strand 3 to strand 4 (β3/β4 loop), and strand 2 to helix H1 (β2//H1 loop) (Fig. 5a). The NMR-derived structure of SrtA differs significantly from the crystal structure of the non-covalent complex between SrtA and the LPXTG peptide (PDB ID 1T2W)^21^, particularly in loops β6/β7 and β7/β8 and in the bound conformation of the LPXTG peptide. The loop β6/β7 was shown to be essential for catalysis, since amino acid mutations in this region impair enzyme activity and alter specific substrate recognition. Furthermore, the NMR spectroscopy data shows that substrate binding induces a structural transition involving loop β6/β7 which transitions from a structurally disordered and open conformation to an ordered and closed conformation. This substantial structural change was not observed in the crystal structure of SrtA non-covalently bound to LPXTG. Molecular docking calculations were performed for Myr using the solution structure of SrtA^22^. Therefore, takes into account the conformational change induced by the substrate, and should be representative of the enzyme intermediate state during catalysis. The molecular docking calculations indicate that Myr binds to the active site groove of SrtA similarly to the covalent bound LPXTG analog (Fig. 5b,c). The resulting lowest energy conformations for the ligand-receptor complex were obtained from extensive sampling of 4.05 × 10^8^ conformations. Remarkably, the 100 lowest energy conformers sampled for Myr are conformationally identical, suggestive of a tight binding of the potential inhibitor into the active site (within a RMSD of 2 Å) (Fig. 5c). Conversely, molecular docking calculations for Myr-gly yield multiple conformations, suggestive of non-productive binding to SrtA compared to Myr.

### Structural model for the Myr-Hla complex

Hla is secreted as a water-soluble monomeric protein, which forms membrane-inserted heptameric pores upon binding to the target bilayer. Considering that hla gene expression was not significantly modulated by Myr and based on our phenotypic assays, which show that Myr suppresses hemolytic activity of *S. aureus* Hla producer supernatants and directly binds to purified Hla preventing hemolysis (Fig. 3g and i, respectively), we have performed computational simulations to investigate the structural basis of Myr-induced inhibition of the Hla oligomerization process. It has been previously shown by means of X-ray crystallography that conformational transitions among the monomeric and heptameric forms of Hla trigger the oligomerization process^23^. Two regions are major players in this process, namely the prestem and amino latch (Supplementary Fig. S4a). In the monomeric Hla the prestem region is folded into a three-stranded antiparallel β-sheet with a long connecting loop next to the cap domain whereas the N-terminal amino latch is located at the edge of the β-sheet of the stem region (Supplementary Fig. S4a). The prestem is fastened to the cap domain with a key hydrogen bond between D45 and Y118 (Supplementary Fig. S4a). Upon oligomerization, the amino latch is released, disrupting the hydrogen bond D45-Y118^23^. This event initiates the protrusion of the prestem characteristic of the Hla heptameric form.

**Figure 6:**
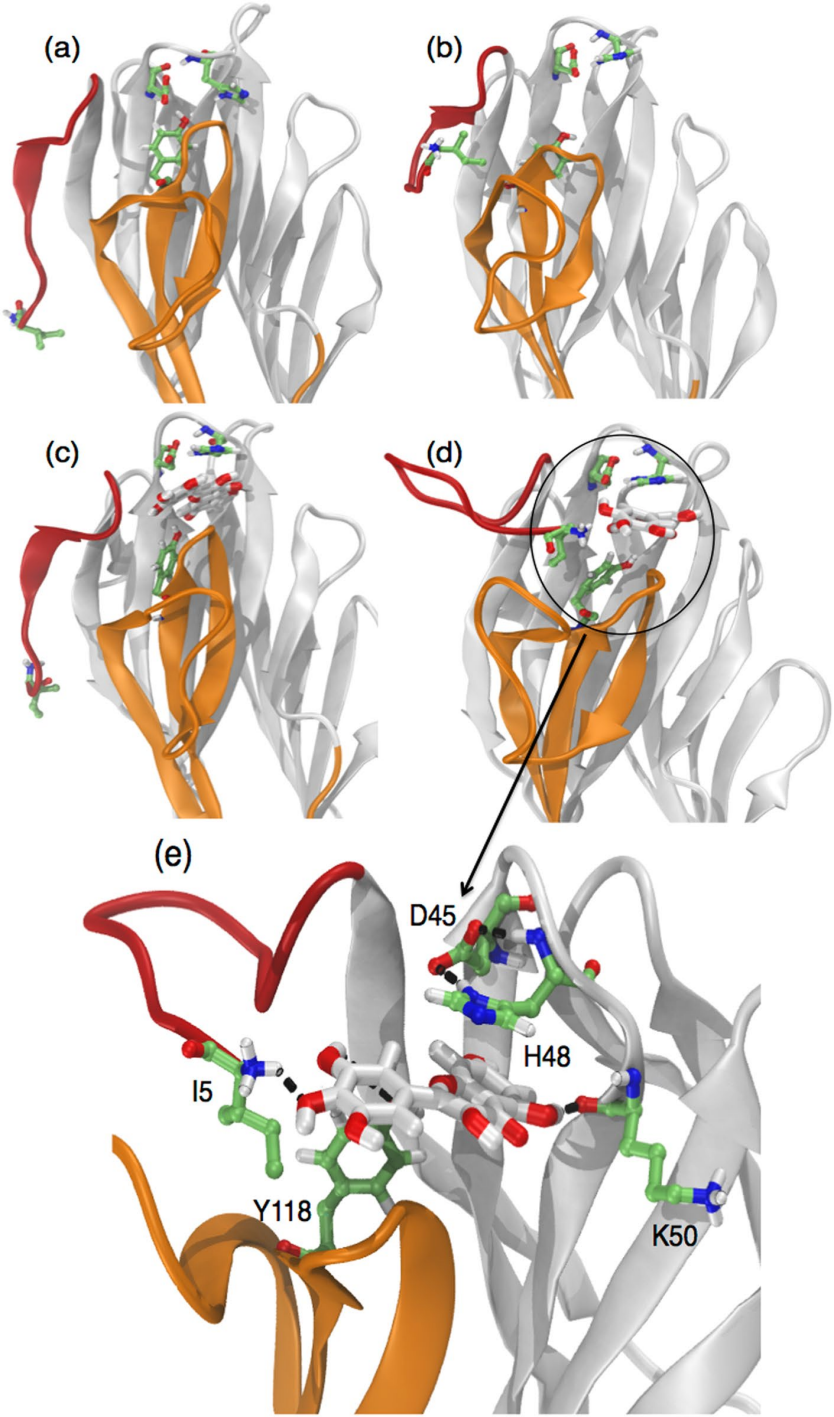
Computational studies of the structural model for the complex Myr-α-hemolysin (Hla). Initial and final conformations of free (**a**,**b**) and Myr-bound (**c**,**d**) Hla obtained from MD simulations in explicit solvent and molecular docking calculations. (**e**) Detailed view of chemical groups and residues involved in the binding of Myr to Hla. The amino latch is colored in red, and the prestem in orange. Receptor residues are shown in green sticks and Myr in white sticks. Hydrogen bonds are represented by black dashed lines. Methodology can be found in Supplementary Methods.

Based on the proposed mechanism of pore formation for Hla, molecular docking calculations were performed for Myr throughout the receptor regions participating in the transition from monomeric to heptameric (pore forming) conformations. As a first approach, molecular docking calculations were directly performed using the X-ray structure of Hla. Extensive conformational sampling for Myr yielded multiple conformations in which Myr bound to the monomeric X-ray structure of Hla with similar binding affinities and without a preferential binding site (Supplementary Fig. S4b). Molecular dynamics (MD) simulations of the wild-type Hla were performed to explore distinct microstates, which could potentially bind Myr in a more specific manner. Our MD simulation shows that the amino latch region switches between a β-strand (in a four-strand β-sheet) and less ordered conformations where the N-terminal residues interact with the prestem region (Fig. 6a,b). During this event, the hydrogen bond D45-Y118 is disrupted. This rupture has been previously postulated to release the amino latch and initiate the protrusion of the prestem to yield the heptameric form of Hla^23^. The latter conformation was subsequently used as initial configuration for another round of molecular docking calculations using Myr as ligand (Fig. 6c). The lowest energy conformation obtained for Myr bound to the MD-derived structure is representative of a cluster of conformers containing 98% of the lowest energy conformers sampled out of 4.05 × 10^8^ possibilities (Fig. 6c). It shows Myr bound to a region of the cap enclosed by the prestem and amino latch regions, in between residues D45 and Y118. The molecular docking calculations suggest that Myr can favorably bind to monomeric Hla with estimated binding energies of ca. 7 kcal.mol^−1^ and K_i_ within the µM range. Subsequently, the Myr- Hla complex was submitted to MD simulations to evaluate the effect of ligand binding on the receptor structural dynamics (Fig. 6c,d). The RMSD calculated for Cα atoms of the complex with respect to the X-ray structure (4YHD) exhibits longer convergence times with increased structural divergence from the crystal structure compared to the free Hla (Supplementary Fig. S5). RMSF profiles for free and Myr-bound Hla are similar but with increased atomic fluctuations for the latter (Supplementary Fig. S6). The most flexible regions occur in the loops containing residues E71-G72, G134-I136 and P160-D162. We examine the consequences of the increased dynamics of these regions upon Hla binding in the Discussion section.

### Prototype of *Green*-coated surface with Myr and surface wettability

**Figure 7:**
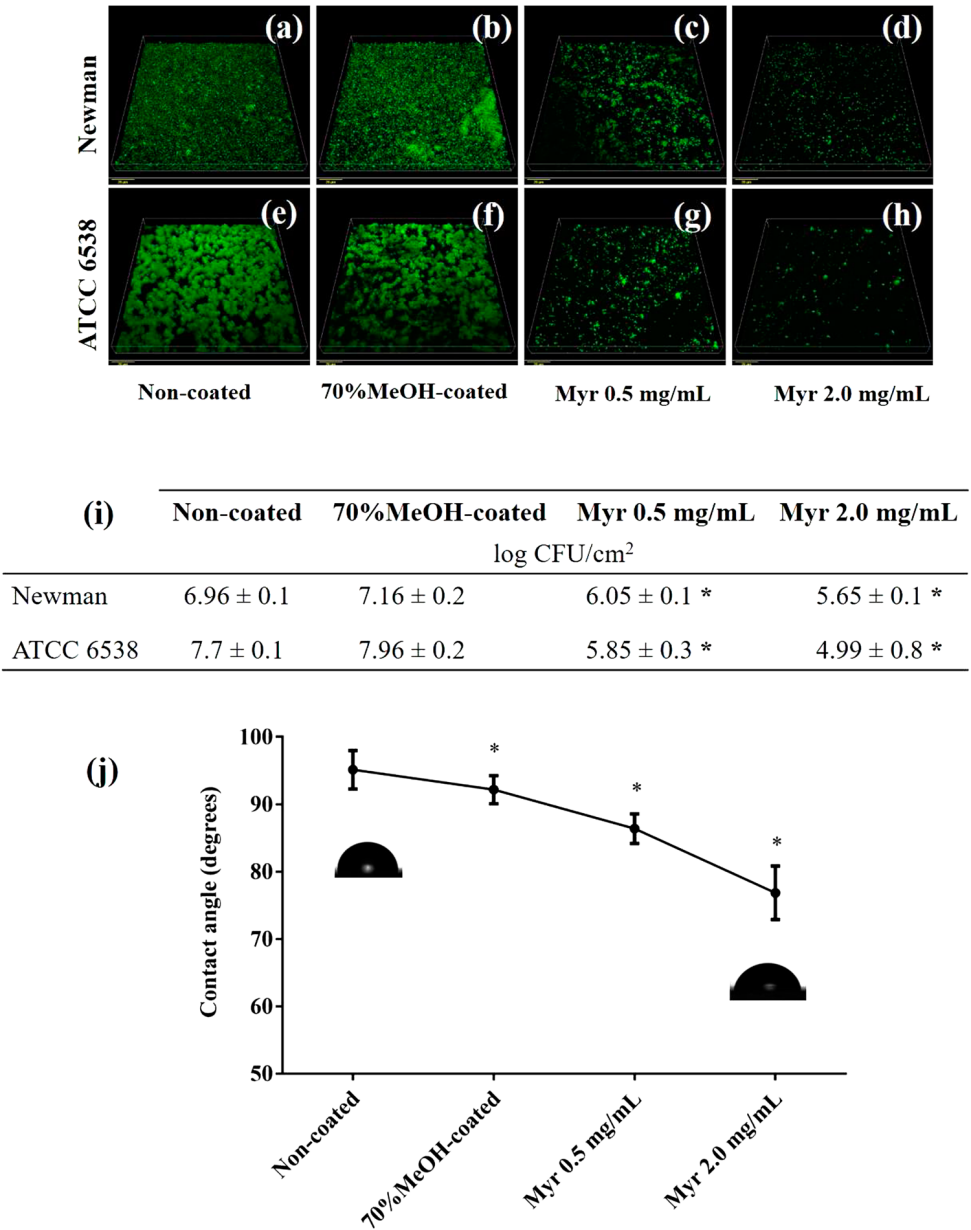
Effects of Myr when coated on a material surface. (**a–d**) Confocal images of *S. aureus* Newman biofilm formation on control surfaces and surfaces coated with Myr. (**e–h**) Confocal images of *S. aureus* ATCC 6538 biofilm formation on control surfaces and surfaces coated with Myr. Bars indicate 20 μm. (**i**) Quantitative data of adhered cells on the surfaces. (**j**) Water contact angle of material surfaces. *Represents statistically significant difference (*p*-value < 0.01) from the non-coated material. All of these methodologies can be found in Supplementary Methods.

Since Myr prevented bacterial adhesion without killing cells, we developed a prototype of a natural product-coated surface, using spin-coating technique, to verify its applicability when immobilized on surfaces. These bioinspired surfaces were strongly resistant to bacterial adhesion presenting only few cell clusters or just single attached cells; thus, resulting in biofilm formation inhibition (Fig. 7c,d and g,h). Non-coated (Fig. 7a and e) and methanol-treated surfaces (Fig. 7b and f) enabled bacterial adherence and accumulation allowing a robust biofilm to form. Colony counting of biofilm scraped from surfaces also evidenced a drop in the number of adhered cells (Fig. 7i). The reduction observed for both strains tested ranged from almost 2–3 log CFU/cm^2^ reduction for *S. aureus* 6538 and 1–1.5 log CFU/cm^2^ for *S. aureus* Newman (Fig. 7i). Moreover, surface characterization indicated that these prototype surfaces had a more hydrophilic character than the non-coated and 70% MeOH-treated Permanox surfaces, presenting a water contact angle of about 76°, 95° and 92°, respectively (Fig. 7j).

### Evaluation of Myr toxicity and protection of infection caused by *S. aureus* in *G. mellonella* larvae model

*Galleria mellonella* larvae is an alternative model to evaluate *in vivo* toxicity and efficacy of new antimicrobial agents^24^. This model represents a quick and economical experimental host to be used prior to more expensive mammalian models. Myr solutions administered to larvae hemocoel, at concentrations up to 50 mg/kg, did not result in death or visible injury, indicating that the flavonoid was not toxic towards the larvae (Supplementary Fig. S3b). This data corroborates with our previous *in vitro* data where we demonstrated that Myr is not toxic towards human erythrocytes (Supplementary Fig. S3a).

**Figure 8:**
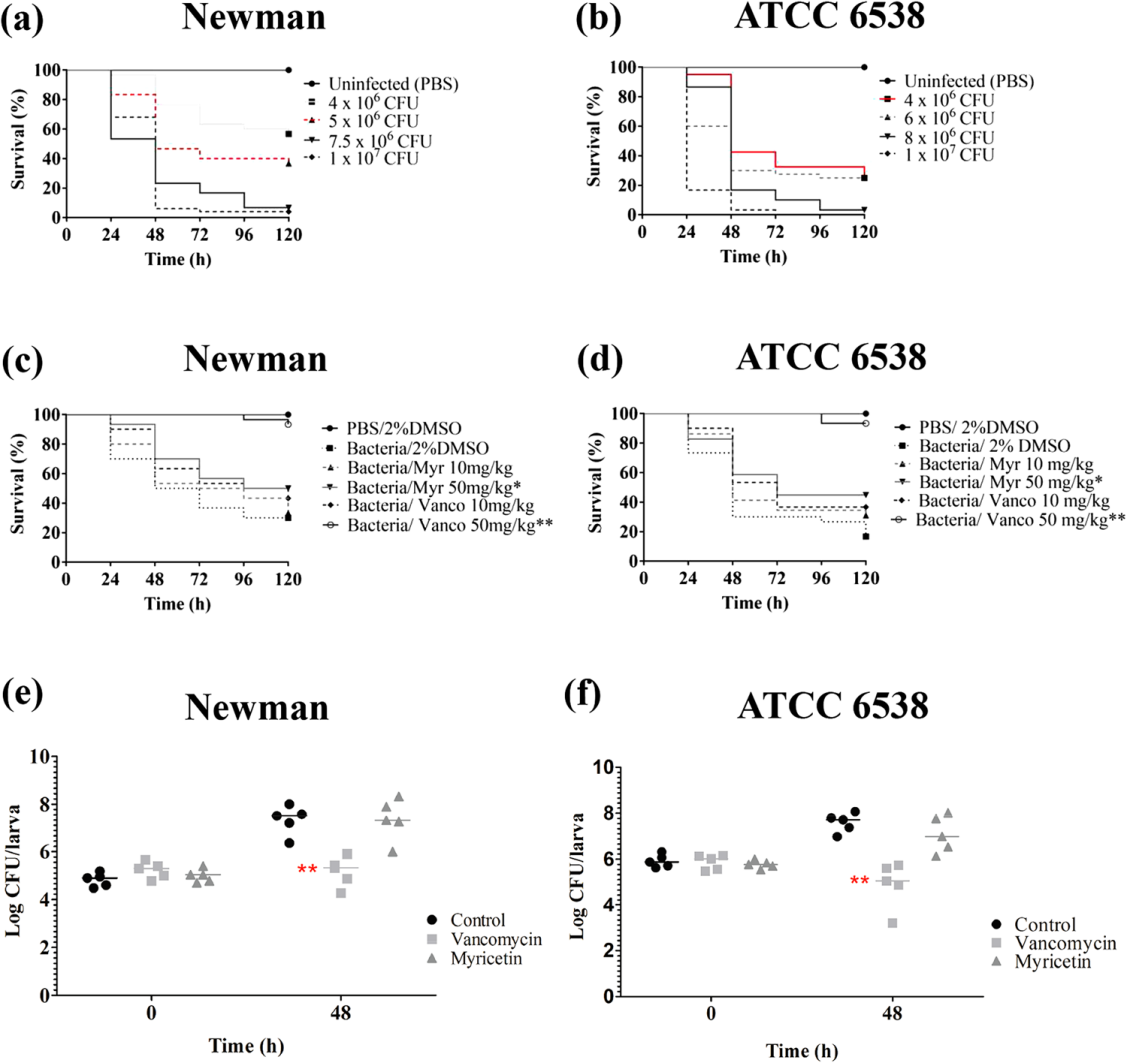
Kaplan-Meier survival-curve of infected *G. mellonella* larva and bacterial burden during infection. (**a**) Survival of *G. mellonella* larvae infected with different concentrations of *S. aureus* Newman. (**b**) Survival of *G. mellonella* larvae infected with different concentrations of *S. aureus* ATCC 6538. The inoculum selected for further experiments are presented in red. (**c**,**d**) Survival of *G. mellonella* larvae infected with *S. aureus* Newman and ATCC 6538, respectively, and treated with two different doses of Myr or vancomycin 30 min post-infection. (**e**,**f**) *S. aureus* Newman and ATCC 6538 larval burden after infection and treatment followed by 0 and 48 h of incubation. Horizontal bars represents the median value of larval burden per group. * And ** represent statistically significant differences, with *p*-value < 0.05 and *p*-value < 0.01, from to the larvae group that was infected and received only PBS.

Regarding the larvae infection, survival curves obtained with different *S. aureus* inoculum concentrations show that larvae survival is reduced with increasing inocula (Fig. 8a,b). Doses of about 5 × 10^6^ and 4 × 10^6^ CFU/larvae were selected for further experiments with *S. aureus* Newman and ATCC 6538, respectively, since they showed significant differences in larval survival compared to the uninfected control group and a gradual reduction in survival rate covering the whole experimental period (Fig. 8a,b, red lines). After infection with *S. aureus*, the groups of larvae treated with Myr or with the positive control vancomycin at 50 mg/kg demonstrated an increased in survival, when compared to the negative control group (Fig. 8c,d). In parallel, larval burden bacterial counts of the Myr-treated group remained similar to the negative control group (PBS-treated) and statistically different from positive control group (vancomycin-treated) (Fig. 8e,f). Vancomycin protected *G. mellonella* from infection in to a greater extent than Myr; it acts as bactericidal agent while Myr exhibits antivirulence effects without modulating growth.

## Discussion

Health agencies worldwide are alarmed by the emergence of multi-drug resistant bacterial pathogens and call for new and effective therapies^25^. In this sense, antivirulence drugs represent potential novel therapeutic alternatives which present distinct action mode compared to classical available antibacterials. Here we show that Myr, a plant derived flavonoid, reduces *S. aureus* virulence in the *in vivo* model of *G. mellonella* without inhibiting bacterial growth (Fig. 8c–f), strongly indicating that Myr exhibits antivirulence properties and displays a distinct mechanism of action than the antimicrobial vancomycin.

The pathogenicity of *S. aureus* is widely related to its ability to produce a large number of virulence factors during different stages of host colonization and infection. The transcriptional control of physiological and virulence genes is synchronized with the changing environmental and nutritional conditions^26^. During initial stages of infection, *S. aureus* preferentially expresses surface proteins that are required for adhesion to host-extracellular-matrix molecules. These surface proteins, referred to as microbial surface components recognizing adhesive matrix molecules (MSCRAMMs), such as fibronectin (Fn)-binding proteins (FnBPs) and clumping factor, are important mediators of early bacterial attachment^7^. Our data show that Myr impair more than 75% of *S. aureus* biofilm formation from initial stages of adhesion with no growth inhibitory activity (Figs 1 and 2). Arita-Morioka, *et al*.^27^ described that Myr inhibits biofilm formation of various *S. aureus* and *Escherichia coli* strains in a dose-dependent manner, also without inhibiting their growth. Although no mechanism was discussed for *S. aureus* inhibition, the authors found that Myr suppresses curli-dependent biofilm formation of *E. coli* by inhibiting cellular functions of the molecular chaperone DnaK. Here we show that Myr modulates surface properties of *S. aureus*, such as fibrinogen clumping (Fig. 3a,b) and hydrophobicity (Supplementary Fig. S2). Moreover, cells treated with Myr display a similar phenotype to untreated cells on Congo Red Agar (Supplementary Fig. S7), suggesting that Myr does not impair *icaADBC*-regulated slime layer formation, also corroborated by the up-regulation of the *ica* operon (Fig. 4).

Indeed, some of the virulence-associated surface proteins are covalently anchored to bacterial cell wall peptidoglycan by the transpeptidase activity of sortases. The sortase A (SrtA) isoform plays a critical role in the establishment of *S. aureus* infection by modulating the ability of the bacterium to adhere to host tissue and abiotic surfaces^13^. Thus, this enzyme comprises a promising pharmacological target that could effectively reduce bacterial virulence^28^. Herein, we have shown that *S. aureus* treated with Myr is characterized by a reduced ability to clump fibrinogen (Fig. 3a,b) and lower surface hydrophobicity (Supplementary Fig. S2), suggesting that Myr modulates surface proteins involved in bacterial adhesion. Since *srt*A, *clf*A and *clf*B expression in bacteria treated with Myr was not significantly different from bacteria treated with Myr-gly, it is likely that a compensatory up-regulation of *fnb*A and *fnb*B genes occurred (Fig. 4). Furthermore, Kang, *et al*.^29^ showed that Myr is capable of inhibiting sortase activity upon specific substrates and sortase-mediated *S. aureus* clumping to fibrinogen. These results led us to investigate a post-transcriptional effect of Myr on the SrtA protein. Computational simulations suggest that Myr adopts a well-defined conformation upon binding to SrtA as opposed to Myr-gly (Fig. 5c). Calculations indicate that Myr anchors to SrtA via hydrogen bonds to residues G119, H120, E165, C184, and π-stacking interactions with R179 (Fig. 5c). The predicted binding mode of Myr is closely related to the binding mode of other previously identified SrtA inhibitors^30^. For instance, the 2-phenyl-2,3-dihydro-1H-perimidine scaffold interacts with SrtA similarly to Myr, and exhibits a IC50 of 47.2 ± 5.9 µM^30^. These calculations suggest that the SrtA can be a molecular target for inhibition by Myr, consistent with our experimental findings.

Expression of *srt*B was significantly up-regulated after Myr exposure, but not in presence of Myr-gly (Fig. 4), indicating a possible modulation on iron acquisition cascade. In accordance with *srt*B up-regulation by Myr, an up-regulation of the *isd*B gene but not of *isd*A was also observed (Fig. 4). This difference in expression may be explained due to the fact that these genes are monocistronic transcripts and under control of different regulators. In fact, it has been stated that *isd*A is under *sig*B control while *isd*B is not^31^ and it is likely that their sensitivity to iron is also different, with *isd*B being more responsive to iron-depletion than *isd*A^32^. Both flavonols (Myr and Myr-gly) were shown to be strong iron chelators (Supplementary Fig. S8b and Supplementary Methods), suggesting that the antibiofilm activity presented by Myr could be related to iron depletion. However, we observed that the addition of iron could not eliminate the antibiofilm effect promoted by Myr and that Myr-gly does not inhibit biofilm formation while also being an iron chelator (Supplementary Fig. S8c,d and Supplementary Methods). Therefore, the inhibition of biofilm formation induced by Myr seems to be iron level-independent and and may rather be explained by the effect of Myr on cell-wall proteins involved in bacterial adhesion process.

During an *S. aureus* infection, the synthesis and secretion of proteins contribute to membrane damage the subsequent invasion of adjacent tissues with α-HL being one of the most well characterized *S. aureus* virulence factors in this respect. Myr did not significantly inhibit *hla* gene expression (Fig. 4) but elicited a superior anti-hemolytic activity when compared with its glycoside (Fig. 3g and h). Moreover, we showed that Myr binds directly to the purified Hla monomer (Fig. 3i), while Myr-gly does not (Fig. 3j), indicating that Myr prevents the formation of the heptameric pore. In fact, our simulations indicate that Myr binds tightly to monomeric Hla (Fig. 6e). It was anchored to the receptor via two highly persistent hydrogen bonds with the carbonyl group of K50 and the hydroxyl group of Y118 throughout the full length of the MD simulation (Supplementary Fig. S9). A third hydrogen bond was formed with the N-terminal region of the amino latch (Supplementary Fig. S9). The latter interaction was brought about by a structural rearrangement of the amino latch, yielding a conformation that resembles an intermediate state between the monomeric and pore protomeric forms of Hla. The structural rearrangement is similar to that seen in the free Hla simulation (Fig. 6a,b); however, while in the latter the amino latch switches reversibly between ordered and less ordered β-strand conformations, the Myr- Hla complex switches irreversibly from the initial β-strand conformation into a loop which remains associated with Myr for the remaining of the simulation time (Supplementary Fig. S9). Myr hampers the reappearance of the D45-Y118 interaction upon binding while keeping the latter residues in place via direct (Y118) or indirect (D45) interactions with the ligand. The present findings suggest that the anti-hemolytic activity of Myr relies on the inhibition of the oligomerization process of Hla via stabilization of an intermediate structural state between the monomeric and pore protomeric forms of the protein. Other reports corroborate our findings, demonstrating that structurally related flavonoids can also bind to Hla and lead to the inhibition of the formation of the heptameric transmembrane pore, which in turn results in a decrease in the host cell damaged induced by the toxin^33, 34^.

Although srtA and hla expression in bacteria treated with Myr or Myr-gly was not significantly different from the control, docking calculations pointed for a direct binding of Myr to these proteins (SrtA and Hla), indicating greater inhibition by Myr than by Myr-gly due to the increased molecular volume of the latter. Additionally, it is likely that there is a greater energy penalty for Myr-gly due to the presence of the very hydrophilic sugar, which could impair the binding of Myr-gly to these proteins.

In addition, we found that Myr-treated cells were more susceptible to H_2_O_2_ killing and presented a reduced staphyloxanthin production (Fig. 3e). Some *S. aureus* strains present deficiency in production of pigment and can be rapidly killed by reactive oxygen species from host neutrophils and fail to form skin abscesses^35^. In this sense, Lee, *et al*.^36^ screened a series of plant flavonoids for staphyloxanthin reduction and pointed flavone, which is the flavonoid backbone, as the most potent inhibitor. It is known that the genes responsible for staphyloxanthin biosynthesis are organized in an operon, *crtOPQMN*, possessing sigma-beta-dependent promoter upstream of *crtO* and a termination region downstream of *crtN* ^37^. We evaluated *crtM* expression, which is involved in the first step of the pigment production: condensation of two molecules of farnesyl diphosphate. Since we observed an up-regulation in *crtM* expression, it is likely that Myr is acting downstream *crtM* gene. Corroborating with this hypothesis, we verified that Myr is not modulating the expression of the promoter *sigB* (Fig. 4).

*Staphylococcus aureus* virulence is controlled by an intricate network of transcription regulators including alternative sigma factor (SigB), DNA binding proteins (SarA and its homologues) and two-component signaling systems (AgrAC, ArlRS, SrrAB, and SaeRS)^38^. Our findings showed that the expression of the global *S. aureus* regulators *rnaIII* (encoded by the *agr* locus), *sar*A and *sig*B was not different between treatments, while *sae*R expression was down-regulated by Myr. The novelty regarding the activity of Myr is based on the fact that it affects both *S. aureus* surface proteins and secreted proteins. This action indicates that Myr antivirulence activity is unrelated to inhibition of the agr system, which was also confirmed by q-RT PCR experiments (Fig. 4). Within this network, Sae appears to be a central downstream regulator that controls the expression of several exoproteins related to adhesion and invasion of host cells^39^. It has been reported that *S. aureus* saeRS mutant resulted in a biofilm-deficient phenotype^40^ and in an attenuated virulence in systemic infections using animal models^41^. Therefore, the inhibitory effect of Myr on *sae*R expression is in accordance with the reduced biofilm formation in vitro and the decreased virulence in *G. mellonella*.

Considering biofilm recalcitrance and the difficulty to eradicate them from medical devices^42^, we generated a prototype material coated with Myr which showed a reduced surface hydrophobicity and was able to strongly prevent *S. aureus* biofilm development (Fig. 7). Corroborating these findings, other reports in literature show that material surfaces presenting a hydrophilic character substantially impair bacterial attachment to abiotic surfaces^43–45^. Although the spin-coating technique is one of the most common methods for applying uniform thin films to substrates, further studies are needed to evaluate a possible grafted version of this polyphenol. Gomez-Florit, *et al*.^46^ recently proposed a bioactive surface based on the covalent immobilization of the flavonoid quercetin, which extended long-term efficacy of the coating and enhanced the soft tissue integration while retaining biological activity. In fact, low immunogenicity and toxicity properties of natural products with recognized therapeutic efficacy, particularly polyphenol-based coatings, represent a *green* alternative to pharmaceuticals^43, 47, 48^.

**Figure 9:**
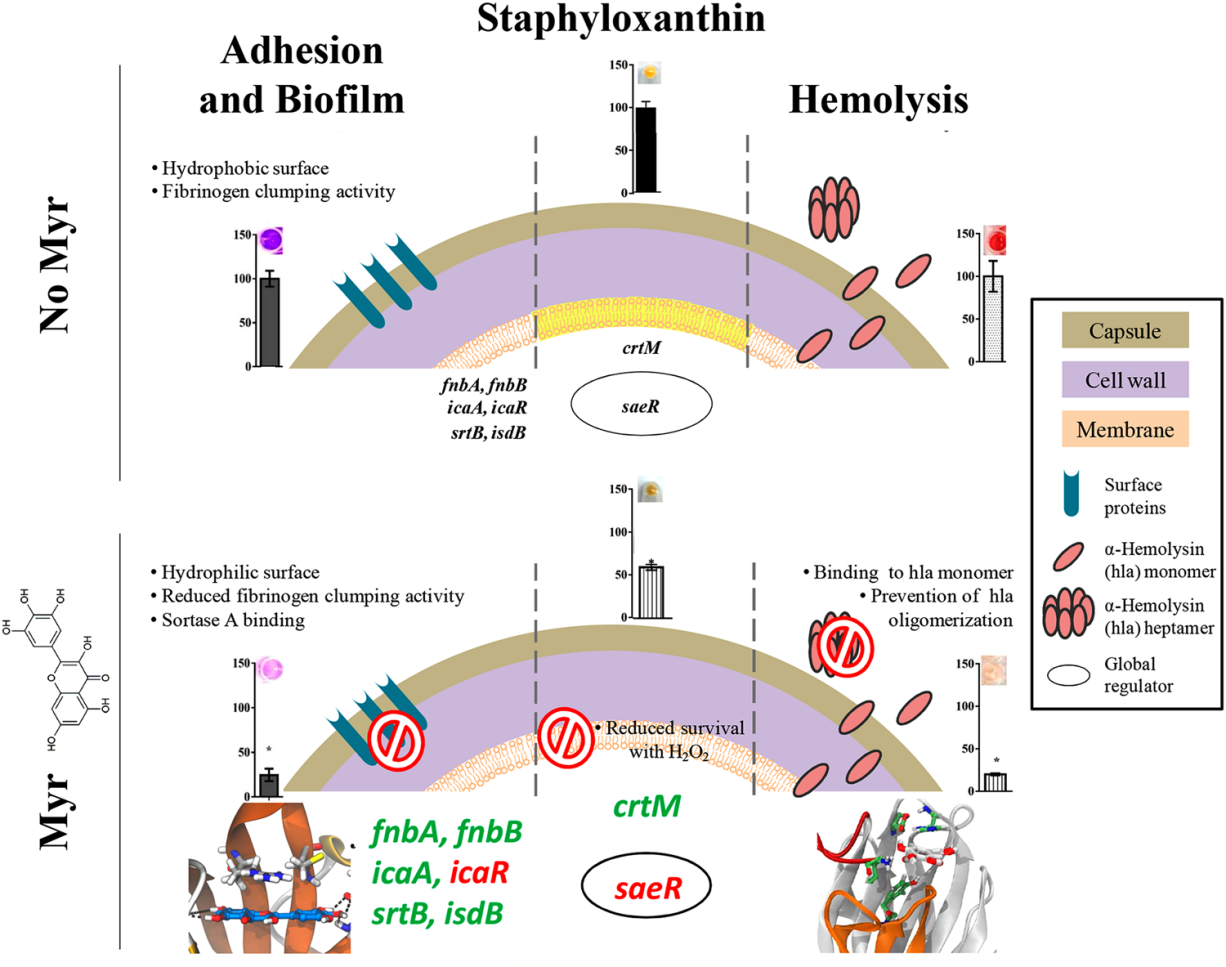
Schematic proposal of the multi-target activity of Myr. Scheme of *S. aureus* virulence factors in absence or presence of Myr, highlighting the multitude of effects of Myr upon adhesion and biofilm formation, staphyloxanthin pigment production and hemolysis induction. Genes names coulored in green and red are representative of up- or down-regulation, respectively.

In summary, the present study highlights the potential of Myr as a multi-target antivirulence agent against the important pathogen *S. aureus*, exhibiting: (i) antibiofilm properties, likely due to the direct binding to the SrtA enzyme and accompanying alteration in expression of cell-wall proteins involved in bacterial adhesion process, allied to the downregulation of the global regulator *sae*R gene; (ii) anti-hemolytic activity due to the direct binding to the Hla toxin, hence preventing the oligomerization process that lead to the formation of the heptameric transmembrane pore in host cells; and (iii) anti-staphyloxanthin activity making *S. aureus* more susceptible to H_2_O_2_ killing (Fig. 9). Moreover, Myr-gly exhibits lower or absence of activity, pointing to the importance of the hydroxyl group in position 3 of the flavonol C-ring for the antivirulence activity of Myr. The net outcome of the effect of Myr on *S. aureus* virulence may be a complex of different activities, including the binding to proteins (SrtA and Hla) and the modulation of at least one global regulator (saeR). As an envisioning of a future practical application, Myr prevented biofilm formation even when immobilized as a thin coating and all effects observed *in vitro* culminated with an attenuated pathogenicity *in vivo*.

## Methods

### Reagents and surfaces

Myricetin (Myr), myricitrin (Myr-gly), quercetin, vancomycin hydrochloride and purified α-Hla were purchased from Sigma-Aldrich (USA). For *in vitro* assays, stock solutions were prepared in dimethyl sulphoxide (DMSO) (Sigma-Aldrich, USA) while for *in vivo* studies, the substances were dissolved in sterile phosphate-buffered salt (PBS) buffer pH 7.0. Sterile 96-well polystyrene flat-bottom microtiter plates (Costar 3599) were purchased from Corning Inc. (USA) and hydrophobic modified polystyrene (Permanox™) slides were purchased from NalgeNunc International (USA). A 10 µL Hamilton® Microliter™ syringe was used to inject inoculum aliquots into *G. mellonella*.

### Bacterial growth kinetics

A kinetic study was performed to assess the effect of the compounds, at concentrations up to 200 µM, on *S. aureus* ATCC 6538 and *S. aureus* Newman growth. The OD_600_ was measured at 0, 1, 2, 4, 6, 8, 12 and 24 h after incubation (37 °C) in BHI broth^49^. Samples were replaced with sterile water as a control for bacterial growth and vancomycin was used as control for bactericide action. The results are expressed as mean ± standard deviation (SD).

### Bacterial strains and culture conditions

*Staphylococcus aureus* Newman ATCC 25904, *S. aureus* ATCC 6538 and *S. aureus* ATCC 29213 were grown in Mueller Hinton (MH) agar (Oxoid Ltd., England). Bacterial suspension in sterile saline or Brain Heart Infusion (BHI) broth (Oxoid Ltd., England), corresponding to optical density at 600 nm (OD_600_) of 0.150 (3 × 10^8^ CFU/mL), was used in the assays.

### Initial adhesion and biofilm formation assays

Initial adhesion and biofilm formation were evaluated using the crystal violet assay in 96-well microtiter plates, as described by Trentin, *et al*.^50^. The incubation period at 37 °C was 1.5, 3, 6 and 24 h. Myr and Myr-gly were tested in concentrations ranging from 5 to 200 µM while untreated control received sterile water in order to correspond to 100% of adhesion or biofilm formation.

### Scanning electron microscopy (SEM)

*Staphylococcus aureus* ATCC 6538 and ATCC Newman biofilms were grown in 96-well microtiter plates (37 °C during 24 h) with a piece of Permanox™ slide. The samples were prepared and examined according to Silva, *et al*.^51^.

### *S. aureus* clumping assay

The clumping assay was performed as previously described by Weiss, *et al*.^14^ with the following modifications. The strains ATCC 6538 and ATCC Newman were cultured in BHI broth during 24 h at 37 °C in the presence of compounds. The cultures were harvested by centrifugation and washed twice with sterile saline. After washings, the pellets were resuspended in a fibrinogen solution consisting of 1 mg/mL of fibrinogen in PBS solution. The absorbance (OD_600_) was measured for each sample 2 h after resuspension in the fibrinogen solution. In the presence of an active sortase protease, clumping factor protein, anchored in the cell wall by sortase, actively recognizes and binds to fibrinogen in solution, forming aggregation or ‘clumps’ which fall out of solution and are traced by a decreasing OD over time. The percent change in absorbance was determined by dividing the absorbance at the time points by that obtained at the initial time point multiplied by 100. We applied quercetin at 200 uM as a positive control for sortase A inhibiton^29^.

### Microbial surface hydrophobicity index

Bacterial surface hydrophobicity was determined using the microbial adhesion to hydrocarbon (MATH) test, according to Trentin, *et al*.^52^. *Staphylococcus aureus* ATCC 6538 and ATCC Newman were cultured in BHI broth during 24 h at 37 °C in the presence of compounds. The cultures were harvested by centrifugation and washed twice with sterile saline solution. The suspensions were adjusted to an absorbance (Ai) of about 0.3 at 600 nm using a spectrophotometer. Toluene (200 μL) was added to 1 mL of each adjusted bacterial suspension and mixed. The new absorbances of aqueous phase (Af) were measured after phase separation. The hydrophobicity index (HPBI) was expressed as: (Ai − Af)/Ai × 100%.

### Staphyloxanthin assay: qualitative and quantitative analysis

The bright golden color of this virulence factor facilitates the anti-virulence screening by the simple observation of color change, however we also applied a quantitative carotenoid evaluation according to Liu, *et al*.^35^, with minor modifications. Briefly, *S. aureus* ATCC 6538 cells were inoculated in BHI medium and incubated for 24 h at 37 °C with or without flavonoids. Bacterial cells were harvested by centrifugation and washed twice with sterile saline and at this point, cell pellets were photographed to compare the staphyloxanthin production. For the extraction of carotenoid pigments, the cell pellets were resuspended in 0.2 mL of methanol by vortexing, and this mixture was heated at 55 °C for 30 min. Pigment extraction was separated from cell debris by centrifugation at 16.600 g for 10 min. The procedure of pigment extraction was repeated 3 times in order to maximize staphyloxanthin extraction, and the optical densities of collected extractions were measured at 465 nm using a spectrophotometer. Each data point was averaged from at least three independent cultures.

### Staphyloxanthin assay: hydrogen peroxide resistance evaluation

The resistance assay (survival test) with hydrogen peroxide (H_2_O_2_) was adapted from a previous study of Liu, *et al*.^35^. *Staphylococcus aureus* ATCC 6538 were cultured in the presence and absence flavonols during 24 h and then harvested by centrifugation and washed twice with sterile saline. Bacterial suspensions were prepared with saline solution in order to obtain a suspension of OD_600_ of 0.150 and incubated with H_2_O_2_ (final concentration of 1.5%) for 60 min at 37 °C with shaking (150 rpm). The percentage of cells surviving the stress was calculated by the counting of the number of colony-forming units (CFU)/mL in comparison with the non-treated control (bacteria exposed to water instead of flavonols and then challenged with H_2_O_2_). The results are the average of at least three independent cultures.

### Hemolysis assay

Firstly, we carried out a simple assay to assess the possible injury caused by flavonoids to human red blood cells. Myr and Myr-gly were tested at 5, 50 and 200 µM, and as reference samples, we used water (for baseline values) and Triton X-100 (for 100% hemolysis). To avoid the interference of sample color, a blank sample of flavonoids and PBS (without erythrocytes) was developed. The assay was calculated as (Abs treatment − Abs blank/Abs Triton − Abs Water) × 100.

Then, the lysis of human red blood cells was measured using *S. aureus* supernants grown in the presence of flavonoids. Briefly, *S. aureus* ATCC 29213, a well-known α-hemolysin producer, was cultured in BHI medium with or without flavonoids during 24 h at 37 °C and 150 rpm. Bacterial supernatants were added to 3% human red blood cell suspensions and were incubated at 37 °C for 1 h at 100 rpm. Supernatants were collected by centrifugation at 3000 × g for 10 min and optical densities were measured at 543 nm. All the blood donors were healthy researchers and students who signed specific form for consent to participate in the study. The Universidade Federal do Rio Grande do Sul Ethical Committee approved all documents, procedures and project under authorization number 1.202.565 (2015).

Adiditionally, we investigate the direct binding of Myr and Myr-gly on purified α-HL. 200 µL PBS plus 1% BSA contening DMSO, Myr or Myr-gly (5, 50 and 200 µM) was preincubated with 200 µL of purified α-HL (125 units of protein- Sigma Aldrich, USA- H9395) at 37 °C for 30 min, followed by the addition of a 1% rabbit erythrocytes suspension in PBS (100 uL). The mixtures were incubated at 37 °C for 30 min. Following centrifugation, the supernatants were removed, and the absorption was measured at 543 nm. Controls without α-HL were performed in order to corrected the effect of color samples. The percent hemolysis was calculated using the supernatant reading of control sample in presence of α-HL (2% DMSO).

### *In vivo* toxicity and survival assay in *Galleria mellonella* larvae

The whole cycle of *G. mellonella* were maintained in our laboratory at 28 °C. Insects were fed with an artificial diet consisting of honey and several flours. Groups of ten larvae of the greater wax moth in the final instar larval stage weighing 220–260 mg were used in all assays. Larvae were injected using a 10 μL Hamilton syringe into the hemocoel in the last right proleg. For toxicity assay, larvae were treated with 10 and 50 mg/kg of Myr. Controls included a group of larvae that did not received any injection and a group of larvae inoculated with vehicle (PBS 2% DMSO).

For infection assay, in order to determine the appropriate concentration of each bacterial strain to be injected in larvae, we performed a curve with different bacterial inoculums ranging from 1 × 10^6^ to 1.0 × 10^7^ CFU/larvae. Then, larvae were infected with 10 uL of bacterial suspension in saline (5 × 10^6^ and 4 × 10^6^ CFU/larvae, respectively for *S. aureus* Newman and ATCC 6538). After 30 minutes of incubation at 37 °C, larvae received 10 µL of flavonoid or vancomycin or vehicle in the last left proleg. Subsequently, all larvae were incubated at 37 °C in sterile petri plates. The following control groups were included: untreated control (larvae not administered any injection), PBS 2% DMSO control (larvae inoculated with vehicle), negative control (larvae inoculated with *S. aureus* and treated with PBS) and positive controls (larvae inoculated with *S. aureus* and treated with vancomycin 10 or 50 mg/kg).

For both toxicity and infection assays, larvae were assessed daily for survival up to 5 days post-treatment and were evaluated according survival, being scored as dead when they displayed no movement in response to touch. Experiments were repeated at least 3 times (10 larvae per group).

### Determination of bacterial burden in *G. mellonella*

According to the protocols modified from Krezdorn, *et al*.^53^ and Richards, *et al*.^54^, groups of 5 larvae were infected with 5 × 10^6^ and 4 × 10^6^ CFU/larvae, respectively for *S. aureus* Newman and ATCC 6538. Myr or vancomycin were administered at 30 min after infection as a single dose. Right after the treatment (time 0 h) and after 48 h, larvae were anaesthetised and surface disinfected with ethanol. The ethanol was poured off and the larvae were allowed to dry. Once dry, each larva was placed into a separate Eppendorf tube containing 1 mL of sterile PBS. A clean pestle was washed in ethanol, flamed and used to homogenise the larva. These homogenate were centrifuged at 2,500 rpm for 5 min at 4 °C, and the liquid phase was retained and kept on ice. Serial dilutions were made and plated onto *Staphylococcus*-selective media (Mannitol salt agar; Merck), and CFU number was determined after a minimum of 48 h of incubation at 37 °C.

### Statistical analysis

Biological assays were carried out at least in triplicate. Data were analyzed by the Student t-test in relation to the untreated samples and *p* ≤ 0.01 was considered to be significant. Survival analysis and statistical significance were determined using the log-rank test and the Kaplan–Meier survival curves (Graphpad Prism 6.0). Significance leval in *G. mellonella* bacterial burden assay was analyzed by Two-way ANOVA with Bonferroni post-test (Graphpad Prism 6.0). qRT-PCR data for target genes with a differential expression of log2 (fold difference) greater than 2 was analyzed by the Student t-test and a significance level of *p* ≤ 0.02 was considered.

## Electronic supplementary material

Supplementary information
