# Supplementary material for: Myricetin protects Galleria mellonella against Staphylococcus aureus infection and inhibits multiple virulence factors
Source: Sci Rep. 2017 Jun 6;7:2823. doi: 10.1038/s41598-017-02712-1 (PMC5460262; doi:10.1038/s41598-017-02712-1)
Supplement: Supplementary file 1 — Supplementary information [file 41598_2017_2712_MOESM1_ESM.doc]

**Supplementary Information**

**Myricetin protects *Galleria mellonella* against *Staphylococcus aureus* infection and inhibits multiple virulence factors**

Silva LN^1,2^, Da Hora, GCA^3^, Soares TA^3,5^, Bojer MS^4^, Ingmer H^4^, Macedo AJ^1,2*^, Trentin DS^1,2^‡

^1^ Centro de Biotecnologia do Estado do Rio Grande do Sul, Porto Alegre, Universidade Federal do Rio Grande do Sul, Porto Alegre-RS, 91501-970, Brazil

^2^ Faculdade de Farmácia, Universidade Federal do Rio Grande do Sul, Porto Alegre-RS, 90610-000, Brazil

^3^ Departmento de Química Fundamental, Universidade Federal de Pernambuco, Recife-PE, 50670-901, Brazil

^4^ Department of Veterinary and Animal Sciences, Faculty of Health and Medical Sciences, University of Copenhagen, Frederiksberg C, 1870, Denmark

^5^ Department of Chemistry, Umeå University, 90187 Umeå, Sweden.

**Present address:**

‡ Departamento de Ciências Básicas da Saúde. Universidade Federal de Ciências da Saúde de Porto Alegre, Porto Alegre-RS, 90050-170, Brazil.

***Corresponding author:**

Dr. Alexandre J. Macedo

Faculdade de Farmácia and Centro de Biotecnologia

Universidade Federal do Rio Grande do Sul, Porto Alegre/RS, Brazil.

E-mail: alexandre.macedo@ufrgs.br

| **Table of contents** | **Page** |
| --- | --- |
| **1. Supplementary Figure S1**: Dose-response curve of Myr tested against *K. pneumoniae* and *P. aerugionsa* biofilm formation and bacterial growth. | **3** |
| **2. Supplementary Figure S2:** *Staphylococcus aureus* surface hydrophobicity index (% HPBI) after treatment with Myr. | **4** |
| **3. Supplementary Figure S3:** Evaluation of Myr and Myr-gly toxicity. | **5** |
| **4. Supplementary Figure S4**: Cartoon representation of the monomeric structure of Hla and the lowest energy conformations of myricetin obtained from molecular docking calculations for the crystal structure. | **6** |
| **5. Supplementary Figure S5**: Root-mean-square deviation of Cα atoms of free and Myr-bound Hla from the X-ray structure as function of time. | **7** |
| **6. Supplementary Figure S6**: Root-mean-square atom-positional fluctuations of Cα atoms of free and Myr-bound Hla from the X-ray structure as function of residue sequence number. | **8** |
| **7. Supplementary Figure S7**: Slime production of *S. aureus* treated with Myr | **9** |
| **8. Supplementary Figure S8**: Iron-chelating assessment of Myr and Myr-gly and its influence on *S. aureus* biofilm formation | **10** |
| **9. Supplementary Figure S9**: Hydrogen bond occurrence map for interactions between Myr and Hla | **11** |
| **10. Supplementary Methods** | **12** |
| 10.1 Isolation of RNA and real-time qRT-PCR of virulence related *S. aureus* genes | **12** |
| 10.2 Computational details | **14** |
| 10.3 Green-coated surfaces: preparation and characterization | **16** |
| 10.4 Green-coated surfaces: confocal microscopy | **17** |
| 10.5 Green-coated surfaces: quantification of adherent bacteria | **17** |
| 10.6 Iron chelating assessment | **17** |
| 10.7 Biofilm formation assay under iron supplementation | **18** |
| 10.8 Congo red agar (CRA) assay | **18** |
| **14. References** | **19** |

**
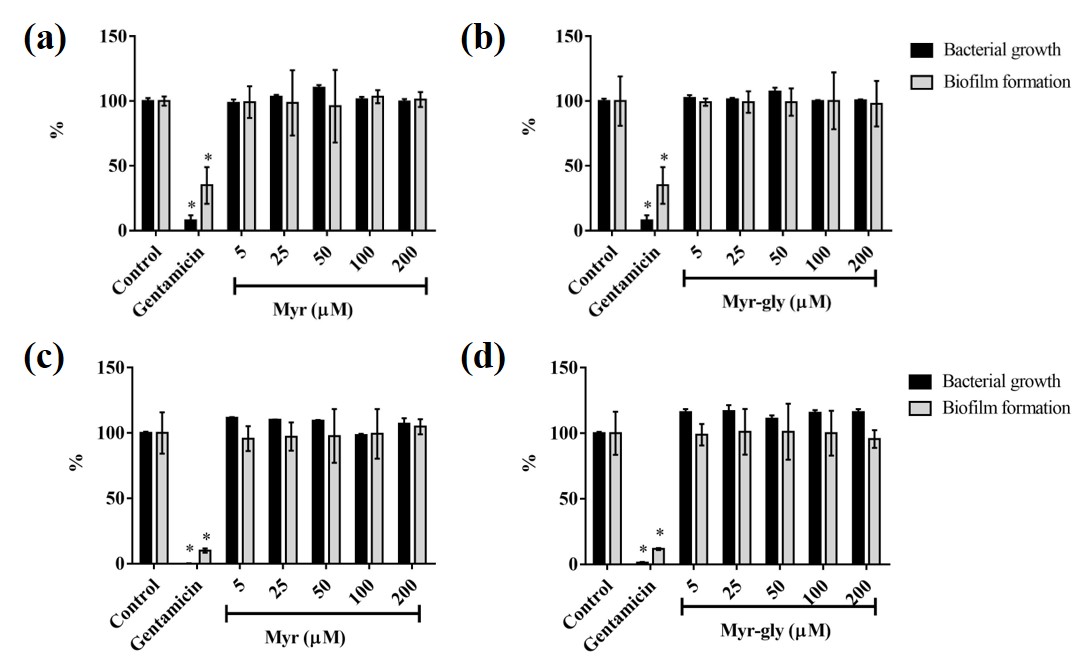
**

**Supplementary Figure S1 Dose-response curve of Myr and Myr-gly tested against *K. pneumoniae* and *P. aeruginosa* biofilm formation and bacterial growth.** Biofilms of *K. pneumoniae* ATCC 700603 exposed to Myr **(a)** or Myr-gly **(b)** and *P. aeruginosa* ATCC 27853 exposed to Myr **(c)** or Myr-gly **(d)** were quantified by the crystal violet staining method and bacterial growth was determined by measuring optical density at 600 nm. * represent statistically significant differences (*p*-value <0.01).

**Supplementary Figure S2 *Staphylococcus aureus* surface hydrophobicity index (% HPBI) after treatment with Myr.** (**a**) Using Newman strain and (**b**) using ATCC 6538 strain. * represents statistically significant differences (*p*-value <0.01) in comparison to the control.

**(b)**

**Supplementary Figure S3** **Evaluation of Myr and Myr-gly toxicity.** (**a**) Evaluation of Myr and Myr-gly cytotoxicity using human red blood cells. 1% Triton X-100 was used as positive control. * represents statistically significant differences (*p*-value <0.01) in comparison to the control. (**b**) Evaluation of Myr toxicity using *G. mellonella* larvae model. Control groups included non-injected larvae and larvae that received 10 uL of PBS vehicle. Myr was dissolved in PBS at 10 or 50 mg/kg and 10 uL of each solution was injected to the larvae proleg. Larvae were monitored according survival up to 120 h at 37°C.

**Supplementary Figure S4** **Cartoon representation of the monomeric structure of Hla and the lowest energy conformations of myricetin obtained from molecular docking calculations for the crystal structure.** (**a**) Representation of Hla domains and regions accordingly to Sugawara and co-workers^1^. The amino latch is colored in red, and the prestem in orange. The cap domain is represented inside a black circle whereas the rim domain is outside. (**b**) Lowest energy conformations obtained for the X-ray structure (4YHD). Receptor residues are represented by green sticks and myricetin by white sticks.


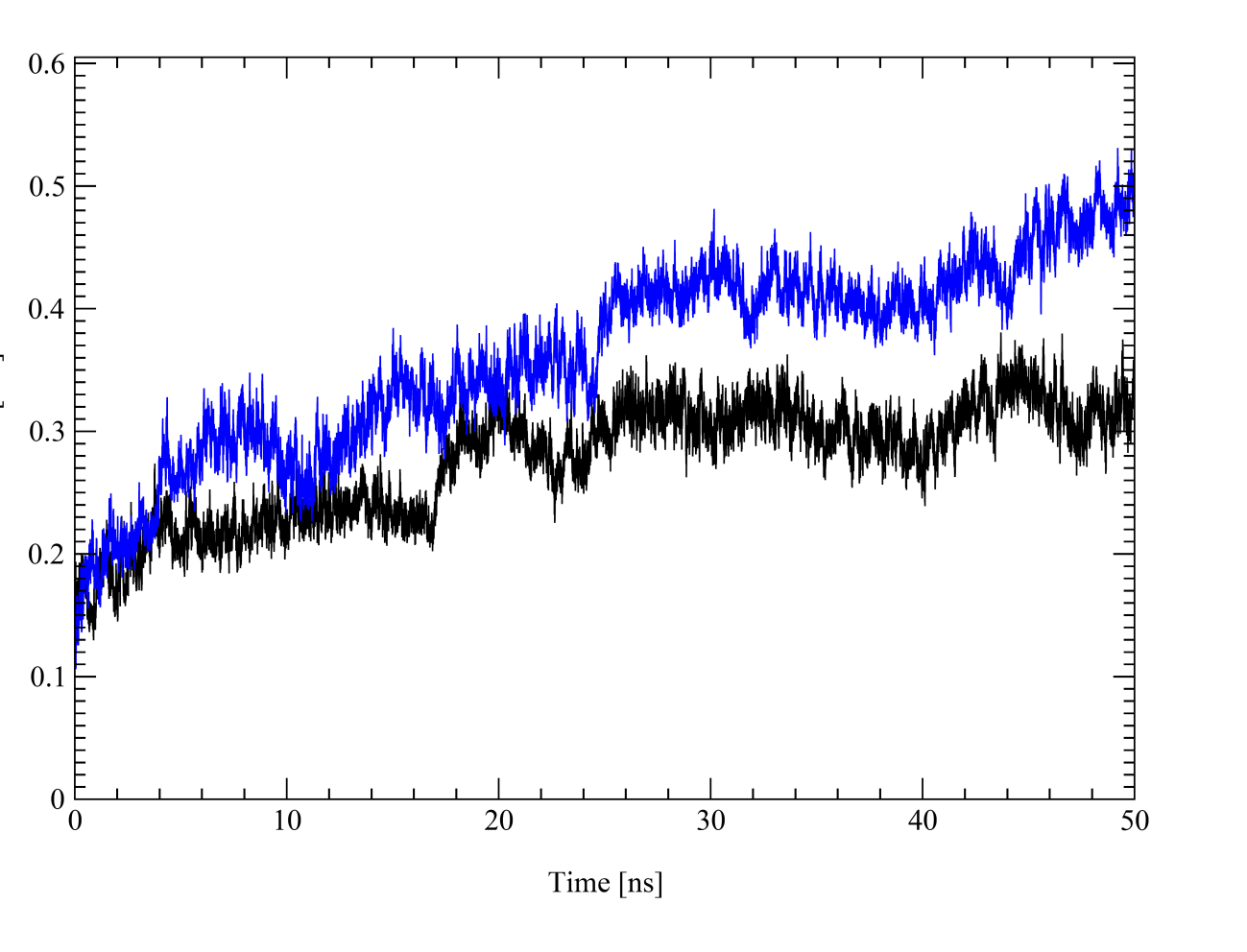


**Supplementary Figure S5** Root-mean-square deviation (RMSD) of Cα atoms of free (black line) and Myr-bound (blue-line) Hla from the X-ray structure (4YHD) as function of time. Rotational and translational fitting of pairs of structures was applied using Cα from residues 15-250 atoms.


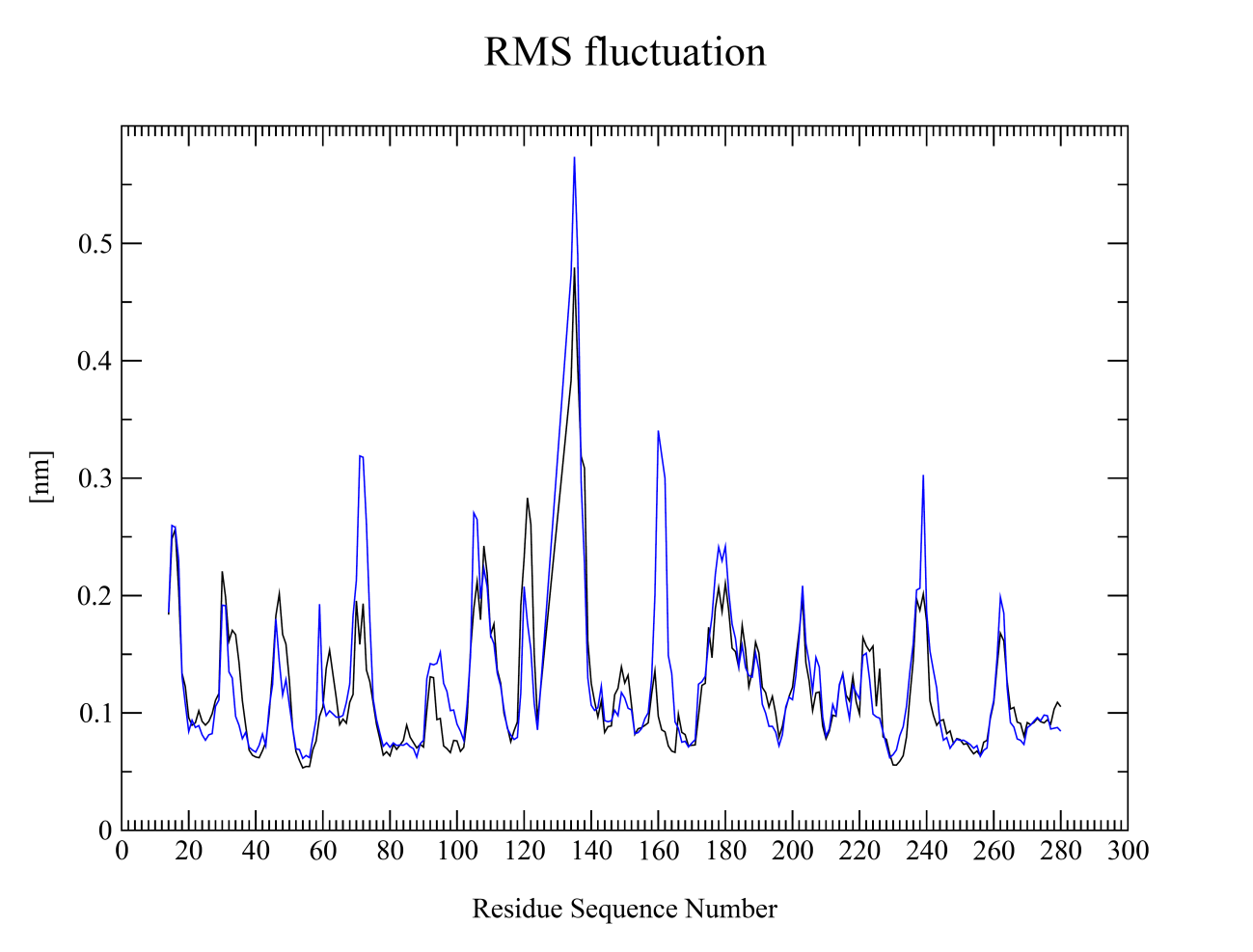


**Supplementary Figure S6** Root-mean-square atom-positional fluctuations (RMSF) of Cα atoms of free (black line) and Myr-bound (blue-line) Hla from the X-ray structure (4YHD) as function of residue sequence number, calculated for the final 2 ns of the two MD trajectories.


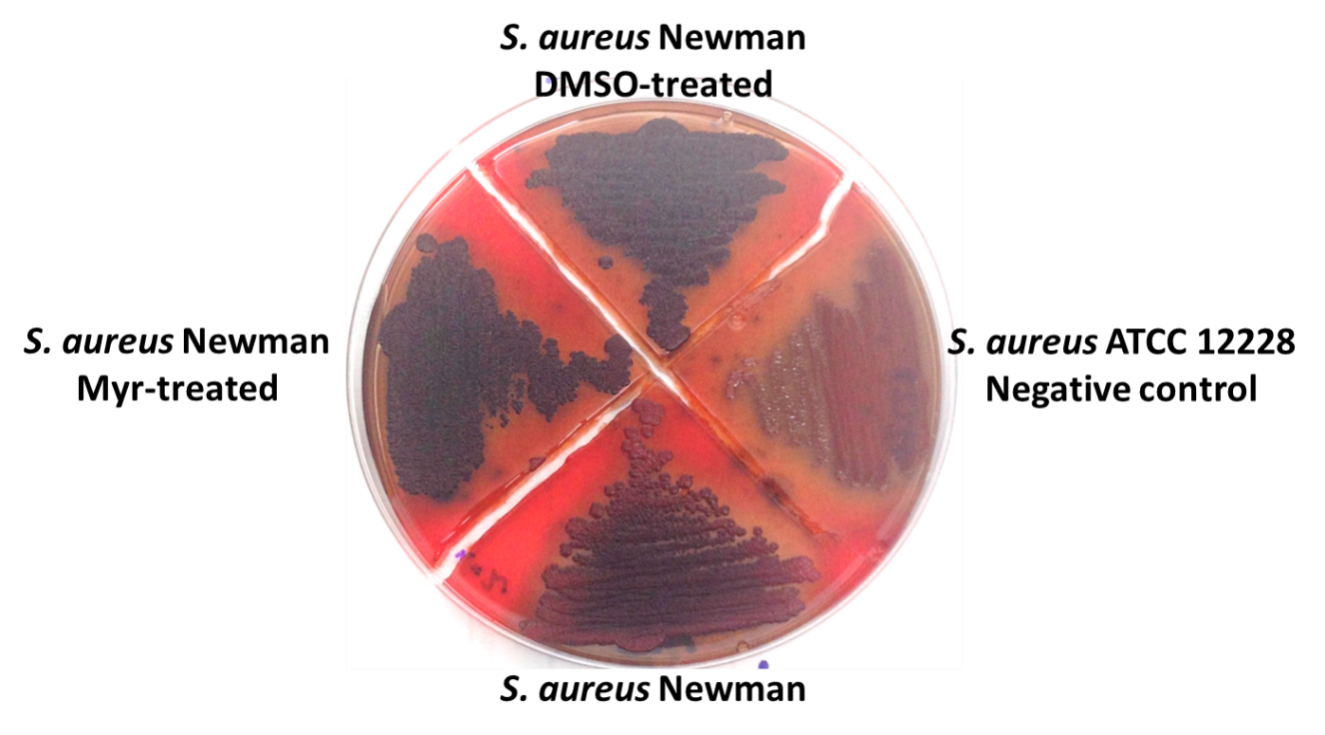


**Supplementary Figure S7 Slime production of *S. aureus* treated with Myr.** Treated cells and control strain were plated on Congo Red Agar in order to detect a modulation on the polysaccharide intercellular adhesin (PIA) production.

**
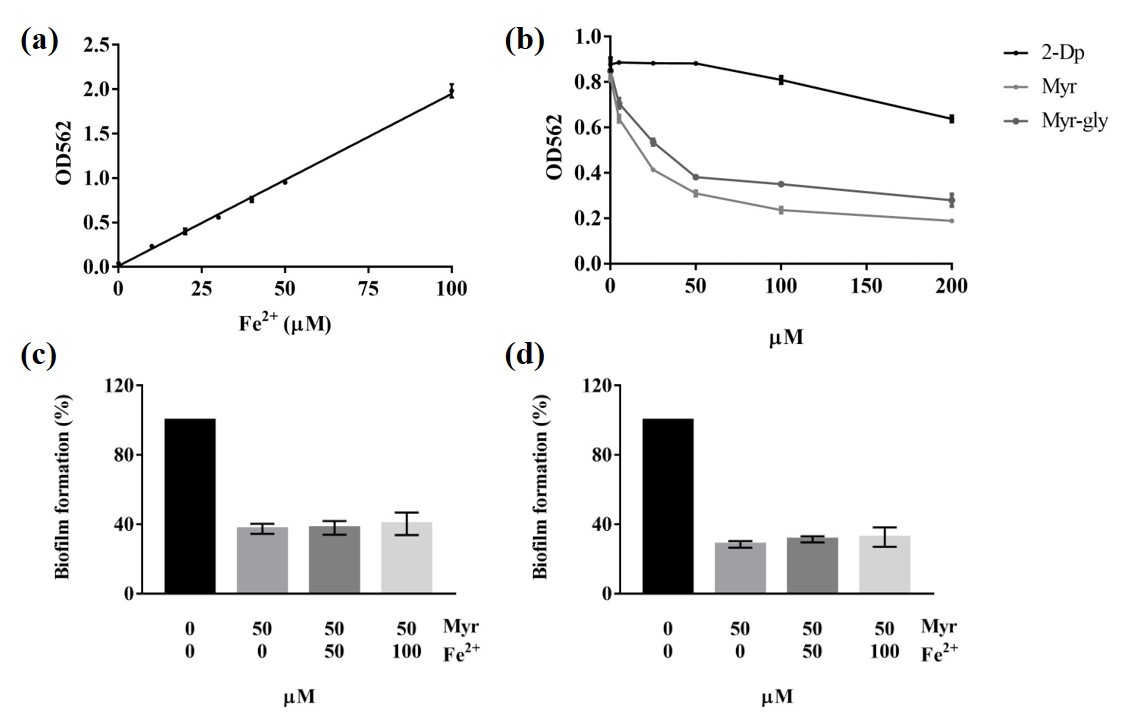
**

**Supplementary Figure S8 Iron-chelating assessment of Myr and Myr-gly and its influence on *S. aureus* biofilm formation.** (**a**) The standard curve established to determine the Fe^2+^ concentration in a ferrozine complex; (**b**) Ferrozine-Fe^2+^ complex quantified in the presence of increasing concentrations of Myr, Myr-gly and positive-chelator 2,2-bipyridyl; (**c-d**) Biofilm formation by *S. aureus* Newman and ATCC 6538 strains, respectively, in the presence of Myr and Fe^2+^ supplementation.


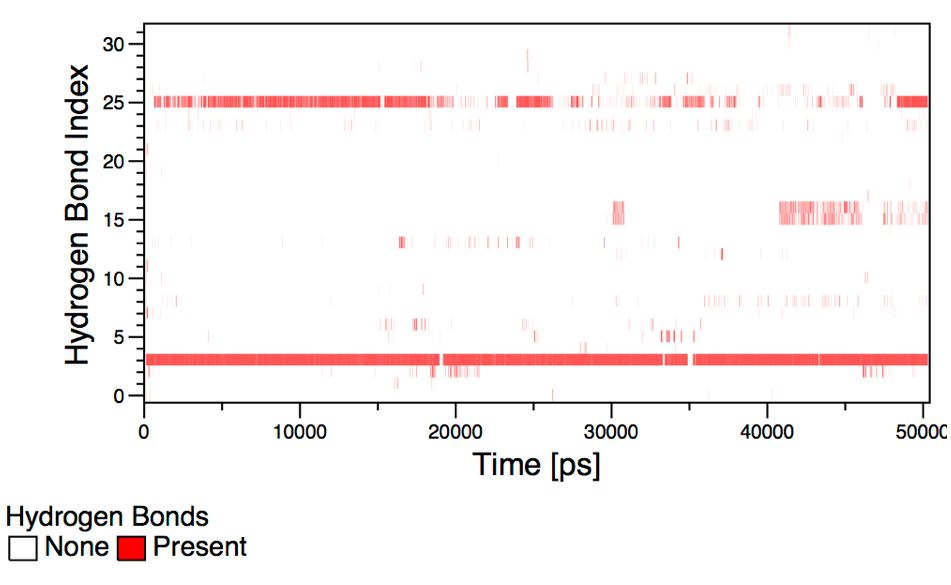


**Supplementary Figure S9 Hydrogen bond occurrence map for interactions between Myr and Hla obtained from explicit solvent MD simulations of the complex**. The hydrogen bond index correspond to hydrogen bonds between hydroxyl groups in the ligand and i. carbonyl group of K50 (index 3), ii. hydroxyl group of Y118 (indexes 25-26) and iii. amino group of I5 (indexes 16-17).

**Supplementary Methods:**

**10.1 Isolation of RNA and real-time qRT-PCR of virulence related *S. aureus* genes**. qRT-PCR was carried out with *S. aureus* Newman grown in presence of 200 µM of Myr, Myr-gly or 2% DMSO treatment as reference using biological triplicates for each condition. The assay was done in BHI with an initial cell density of OD = 0.05 and samples taken after 2.5 h (with an OD of about 3.0) whereafter bacterial cell pellets were frozen at -80°C. RNA was purified from cell pellets using the RNeasy kit (Qiagen) as per manufacturer's instructions with an initial FastPrep-mediated lysis of the cells. Genomic DNA was removed from the samples by treatment with 1U DNase I, RNase-free (Thermo Scientific) for 60 min. at 37°C. Conversion into cDNA was performed with the High-Capacity cDNA Reverse Transcription Kit (Applied Biosystems) including controls to which no reverse transcriptase enzyme was added. Samples of cDNA were diluted 1/20 and 5 µl assayed in each real-time qPCR reaction (FastStart Essential Green Master and a LightCycler® 96 instrument, both Roche) using primers listed in Supplementary Table S1 and technical duplicates for each target. Results were calculated using the comparative cycle threshold method, in which the amount of target mRNA was normalized to that of the two reference genes *ileS* and *pyk*.

**Supplementary Table S1** **Target genes and primers used for RT-qPCR.**

| **Target primers** | **Oligonucleotide (5'-3')** |
| --- | --- |
| icaA_F | AGCGAAGTCAGACACTTGCTGG |
| icaA_R | CAGTATCCCAGTAGCCAACGTCG |
| icaR_F | AGGGGTATGACGGTACAACACTTG |
| icaR_R | TTTCGAGTCAAAATGGTAATATAAACTCGC |
| saeR_F | GCCTTAACTTTAGGTGCAGATGACTATGTC |
| saeR_R | CGACAGTTGTTCAACTGGTTGATGATGG |
| srtA_F | TATATCCAGGACCAGCAACACCTGAAC |
| srtA_R | ACGGTCAATGAAAGTGTGTCCTGC |
| srtB_F | GATTTTGAGCGAGAACATCGACGTAAAGG |
| srtB_R | CGTATTATCACCGACATGGTGCCC |
| fnbA_F | AAGCACCAAAAGCAGTACAAGCACC |
| fnbA_R | TGTGTTGTTTCCTTAACTTGAGGTTTCGC |
| fnbB_F | GAGTTCAGCTACTGAAAGTAAAGCAAGCG |
| fnbB_R | TGATGGTTGCTCAGTTGATGTCGC |
| clfA_F | CACTAATAATGGCGAAACGAGTGTGGC |
| clfA_R | GTCGTAGTAGTAGCTTCACCAGTTACCG |
| clfB_F | ATGCTAATAGCATAGCAACAAACAGTGAGC |
| clfB_R | GCATTTACTACCGGTTCAGCAACAGC |
| sigB_F | GTCCTTTGAACGGAAGTTTGAAGCC |
| sigB_R | CGTCTCGGAACATGTACACTCCAAG |
| sarA_F | GAGTTGTTATCAATGGTCACTTATGCTGAC |
| sarA_R | TCGCTGATGTATGTCAATACAGCGAATTC |
| isdA_F | CAGACAGCCAACAAGTCAATGCG |
| isdA_R | GTCATCCATGTGTGACTTCTCTGAAGAGC |
| isdB_F | GAAGTTAAACCAGCAGCAAAAGCCAC |
| isdB_R | GGACGAGAGTTTGGTGCGCTATG |
| crtM_F | GGTGTTGCTGGTACAGTAGGTGAAG |
| crtM_R | GCAACGATTCACCAAGTCTTCTTGCG |
| hla_F | CTGTCGCTAATGCCGCAGATTCTG |
| hla_R | CTTCTTCGCTATAAACTCTATATTGACCAGC |
| rnaIII_F | GCACTGAGTCCAAGGAAACTAAC |
| rnaIII_R | AAGCCATCCCAACTTAATAACC |
| ileS_F | ACATACAGCACCAGGTCACG |
| ileS_R | CGCCTTCTTCAGTAAATACACC |
| pyk_F | AGGTTGAACTCCCCAAACAA |
| pyk_R | GCAGCCCAAGATTACAAAAA |

**10.2 Computational details.** Atomic coordinates for Hla were taken from the crystallographic structure of the monomeric form solved at 2.8 Å from the sequence of *S. aureus* (PDB ID 4YHD)^1^. The X-ray structure was obtained for the single mutant H35A. The mutation was reversed to the wild-type sequence prior the docking and molecular dynamics (MD) simulations. Missing residues (129-TGKIGGLIG-137) in the X-ray structure were homology modeled using the X-ray structure of the Hla heptamer (PDB ID 3ANZ)^2^ as target. Molecular docking calculations were also performed for Sortase A using the X-ray structure solved at 2.8 Å from the sequence of *S. aureus* (PDB ID 1T2W)^3^. All the structural modeling was performed with the SWISS-MODEL software^4^.

Molecular docking calculations were performed using the Autodock 4.2^5–7^ and AutoGrid4^8^ software combined with the AutoDock Tools^9^. Partial charges for the receptor atoms were assigned according to AMBER86 force field parameters^10^ while ligand charges were calculated with the Gasteiger method^11^. Dihedral angles were treated as fully flexible for ligands. Grid resolution and center were adjusted to each one of the systems. Grid maps of 126 x 126 x 126 points, point spacing of 2.5 Å and centered at 31.156 13.82 -31.295 Å were used for Hla prior to MD simulations. After the MD simulations, grid maps of 122 x 122 x 124 points, point spacing of 2.5 Å and centered at 68.492 44.987 68.103 Å were used. For Sortase A, grid maps with dimensions of 126 x 126 x 126 Å and point spacing of 1.4 Å were centered at 34.798 -14.795 3.594 Å. The Lamarckian genetic algorithm was used with the following parameters: 150 random individuals in an initial population, a maximum number of 2500000 energy evaluations, a maximum number of 27000 generations with mutation and crossover rates of 0.02 and 0.08, respectively. An optional elitism parameter equal to 1 was applied, determining the number of top individuals that will survive into the next generation. A maximum of 300 iterations per local search was allowed. The probability of performing a local search on an individual was 0.06 where the maximum number of consecutive successes or failures before doubling or halving the search step was 4. A total of 100 LGA runs were performed. After the conformational search, docked conformations were sorted in order of increasing energy. The coordinates of the lowest energy conformation were clustered based a root-mean-squared-deviation of 2.0 Å^5–7^. A more detailed description of the methodology employed has been previously presented^12,13^.

MD simulations were performed for the free and Myr-bound Hla receptor. Optimized atomic coordinates and atomic parameters for Myr were obtained from the Automated Topology Builder Repository version 2.2^14^. The GROMOS force field parameter set 54A7 was used in the MD simulations^15^. The simulations were performed in explicit solvent using Single Point Charge (SPC) model^16^ in a cubic box of 10.0 x 10.0 x 10.0 nm3. Periodic boundary conditions were applied in all directions. The system was neutralized with 3 Cl- counter ions described by the GROMOS parameter set 53A6^17,18^. The systems were first energy-minimized for 5000 steps. Under NPT conditions, a time step of 0.001 ps was applied during the equilibration and production phases. Center of mass motion was removed at every 5 steps. The temperature of 298 K was maintained by using the Berendsen thermostat^19^, coupling separately the temperatures of the protein and the solvent via a time constant of 0.2 ps for each. The pressure was maintained by weakly coupling the particle coordinates and box dimension to an isotropic pressure bath at 1.0 bar. The relaxation time was 0.1 ps and a compressibility of 4.5 × 10^−5^ (kJ mol^−1^ nm^−3^)^−1^ as appropriate for water[^64^](#_ENREF_64). The generalized reaction field was applied to treat long-range electrostatic interactions with a dielectric constant of 66^20^. A cutoff of 1.4 nm was used for both van de Waals and long-range interactions. The pair list for short-range non-bonded and long-range electrostatic interactions was updated with a frequency of 10 fs for all simulations. Configurations of the trajectory were recorded every 0.5 ps.

All MD simulations and analyses were performed with GROMACS v.4.6.7^21^. Coordinates and trajectories were visualized with the software VMD version 1.9.1^22^.

**10.3 Green-coated surfaces: preparation and characterization.** Permanox™ surface was coated as previously described by [Trentin, et al.](#_ENREF_22) ^23^. Precisely 200 μL of a 0.5 mg/mL and 2.0 mg/mL in 70% aqueous metanol (Merck, Germany) was spin-coated onto a 1 cm^2^ fragment of Permanox™ during a cycle of 500 rpm (5s) and then accelerated to 5000 rpm (40 s) in the spin coater Laurell Model WS-650MZ-23NPP/LITE. After this first coating step, the specimens were heat-treated (30 min at 60°C) to allow for film annealing and to remove any excess solvent. The coating process was repeated twice and the second coating step was followed by 1h 30 min anneling at 60°C. Samples with coating film double were sterilized with UV light during 20 minutes. As controls, samples were spin-coated with 70% aqueous methanol solution but without Myr and other samples without coating were just heated to 60°C and UV-treated.

Permanox™ samples were characterized before and after coating with Myr using water contact angle (WCA). Contact angle measurements were carried out using the sessile drop technique and milliQ water. The drop was observed directly using an Optical Tensiometer Theta Lite (OneAttension, Biolin Scientific, Finland). The reported water contact angles are means of more than five measurements performed on different areas of each sample surface.

**10.4 Green-coated surfaces: confocal microscopy.** Slides of the non-coated and coated Permanox™ were placed in the wells of 24-well tissue culture plates wherein biofilms were grown (37°C during 24 h). After, the slides were gently washed with PBS and were stained with LIVE/DEAD BacLight Bacterial Viability Kit (Life Technologies, USA). Images were obtained using an Olympus IX81 confocal microscope and UPLSAPO 60X W NA:1.20 objective and were overlaid using Image J software.

**10.5 Green-coated surfaces: quantification of adherent bacteria.** Slides of the non-coated and coated Permanox™ were placed in the wells of 24-well tissue culture plates wherein biofilms were grown (37°C during 24 h). After, the slides were gently washed with PBS and transferred to tubes containing 2 mL of sterile saline. The tubes were submitted to a cycle of 30 seconds of vortex plus 2 minutes of sonication plus 30 seconds of vortex to remove bacterial biofilm from slides. Serial dilutions of each tube content were performed and were spread on MH agar plates. After overnight incubation at 37 °C, the number of colony-forming units (CFU) was determined and expressed as log CFU/cm^2^.

**10.6 Iron chelating assessment.** To evaluate whether iron chelation could account for Myr activity against *S. aureus* biofilm formation, the ferrozine assay was performed using Fe^2+^ as source of iron^24^. A standard curve was established to determine the Fe^2+^ concentration to be used in the ferrozine assay (Supplementary Fig. S8a). Formation of the iron-ferrozine complex was measured at 562 nm to assess the amount of iron chelated by Myr and Myr-gly. For comparison of iron-chelating activity, 2,2-bipyridyl (Sigma-Aldrich Co., USA) was used as a standard iron chelator.

**10.7 Biofilm formation assay under iron supplementation.** *S. aureus* strains were cultured in BHI broth in the presence of Myr (50 µM) plus Fe^2+^ (50 or 100 µM) in 96-well microtiter plates at 37°C for 24 h. The amount of biofilm formation was determined by crystal violet and the sample that was not treated with Myr or Fe^2+^ was set as 100% biofilm formation.

**10.8 Congo red agar (CRA) assay.** Colony morphologies and phenotypic changes were investigated using CRA, as previously described with some modifications^25^. The medium was composed of 37g/L brain–heart infusion broth (BHI) 36 g/L of sucrose, 15 g/L of agar, and 0.8 g/L of congo red. Briefly, *S.* *aureus* Newman were treated with Myr or 2% DMSO during 24 h at 37 °C, then plated on CRA and aerobically incubated for 24 h. *S. epidermidis* ATCC 12228 was used as negative slime producer (negative control). Positive result for slime production was indicated by black colonies with a dry crystalline consistency, indicating polysaccharide intercellular adhesin (PIA) production, while non-slime producing strains usually remain pink.

**References:**

1. Sugawara, T. *et al.* Structural basis for pore-forming mechanism of staphylococcal α-hemolysin. *Toxicon* **108,** 226–231 (2015).

2. Tanaka, Y. *et al.* 2-Methyl-2,4-pentanediol induces spontaneous assembly of staphylococcal alpha-hemolysin into heptameric pore structure. *Protein Sci.* **20,** 448–456 (2011).

3. Zong, Y., Bice, T. W., Ton-That, H., Schneewind, O. & Narayana, S. V. L. Crystal structures of *Staphylococcus aureus* sortase A and its substrate complex. *J. Biol. Chem.* **279,** 31383–31389 (2004).

4. Biasini, M. *et al.* SWISS-MODEL: modelling protein tertiary and quaternary structure using evolutionary information. *Nucleic Acids Res.* **42,** W252-8 (2014).

5. Morris, G. M. *et al.* Automated docking using a Lamarckian genetic algorithm and an empirical binding free energy function. *J. Comput. Chem* **19,** 1639–1662 (1998).

6. Huey, R., Morris, G. M., Olson, A. J. & Goodsell, D. S. A semiempirical free energy force field with charge-based desolvation. *J. Comput. Chem.* **28,** 1145–1152 (2007).

7. Morris, G. M. *et al.* AutoDock4 and AutoDockTools4: Automated docking with selective receptor flexibility. *J. Comput. Chem.* **30,** 2785–2791 (2009).

8. Goodford, P. J. A computational procedure for determining energetically favorable binding sites on biologically important macromolecules. *J. Med. Chem.* **28,** 849–857 (1985).

9. Sanner, M. F. Python: a programming language for software integration and development. *J. Mol. Graph. Model.* **17,** 57–61 (1999).

10. Weiner, S. J., Kollman, P. A., Nguyen, D. T. & Case, D. A. An all atom force field for simulations of proteins and nucleic acids. *J. Comput. Chem.* **7,** 230–252 (1986).

11. Gasteiger, J. & Marsili, M. Iterative partial equalization of orbital electronegativity—a rapid access to atomic charges. *Tetrahedron* **36,** 3219–3228 (1980).

12. Soares, T., Goodsell, D., Ferreira, R., Olson, A. J. & Briggs, J. M. Ionization state and molecular docking studies for the macrophage migration inhibitory factor: the role of lysine 32 in the catalytic mechanism. *J. Mol. Recognit.* **13,** 146–156 (2000).

13. Soares, T. A., Goodsell, D. S., Briggs, J. M., Ferreira, R. & Olson, A. J. Docking of 4-oxalocrotonate tautomerase substrates: implications for the catalytic mechanism. *Biopolymers* **50,** 319–328 (1999).

14. Koziara, K. B., Stroet, M., Malde, A. K. & Mark, A. E. Testing and validation of the Automated Topology Builder (ATB) version 2.0: prediction of hydration free enthalpies. *J. Comput. Aided. Mol. Des.* **28,** 221–233 (2014).

15. Schmid, N. *et al.* Definition and testing of the GROMOS force-field versions 54A7 and 54B7. *Eur. Biophys. J.* **40,** 843–856 (2011).

16. Berendsen, H. J. C., Postma, J. P. M., van Gunsteren, W. F. & Hermans, J. in *Intermolecular Forces: Proceedings of the Fourteenth Jerusalem Symposium on Quantum Chemistry and Biochemistry Held in Jerusalem, Israel, April 13--16, 1981* (ed. Pullman, B.) 331–342 (Springer Netherlands, 1981). doi:10.1007/978-94-015-7658-1_21

17. Oostenbrink, C., Soares, T. A., van der Vegt, N. F. A. & van Gunsteren, W. F. Validation of the 53A6 GROMOS force field. *Eur. Biophys. J.* **34,** 273–284 (2005).

18. Oostenbrink, C., Villa, A., Mark, A. E. & van Gunsteren, W. F. A biomolecular force field based on the free enthalpy of hydration and solvation: the GROMOS force-field parameter sets 53A5 and 53A6. *J. Comput. Chem.* **25,** 1656–1676 (2004).

19. Berendsen, H. J. C., Postma, J. P. M., van Gunsteren, W. F., DiNola, A. & Haak, J. R. Molecular dynamics with coupling to an external bath. *J. Chem. Phys.* **81,** (1984).

20. Tironi, I. G., Sperb, R., Smith, P. E. & van Gunsteren, W. F. A generalized reaction field method for molecular dynamics simulations. *J. Chem. Phys.* **102,** (1995).

21. Hess, B., Kutzner, C., van der Spoel, D. & Lindahl, E. GROMACS 4: Algorithms for highly efficient, load-balanced, and scalable molecular simulation. *J. Chem. Theory Comput.* **4,** 435–447 (2008).

22. Humphrey, W., Dalke, A. & Schulten, K. VMD: Visual molecular dynamics. *J. Mol. Graph.* **14,** 33–38 (1996).

23. Trentin, D. S. *et al.* Natural green coating inhibits adhesion of clinically important bacteria. *Sci. Rep.* **5,** 8287 (2015).

24. Mladenka, P. *et al.* In vitro analysis of iron chelating activity of flavonoids. *J. Inorg. Biochem.* **105,** 693–701 (2011).

25. Freeman, D. J., Falkiner, F. R. & Keane, C. T. New method for detecting slime production by coagulase negative staphylococci. *J. Clin. Pathol.* **42,** 872–874 (1989).
